# Tight association of autophagy and cell cycle in leukemia cells

**DOI:** 10.1186/s11658-022-00334-8

**Published:** 2022-04-05

**Authors:** Alena Gschwind, Christian Marx, Marie D. Just, Paula Severin, Hannah Behring, Lisa Marx-Blümel, Sabine Becker, Linda Rothenburger, Martin Förster, James F. Beck, Jürgen Sonnemann

**Affiliations:** 1 Department of Pediatric Hematology and Oncology, Children’s Clinic, Jena University Hospital, Jena, Germany; 2 Research Center Lobeda, Jena University Hospital, 07747 Jena, Germany; 3 Leibniz Institute on Aging-Fritz Lipmann Institute (FLI), 07747 Jena, Germany; 4 Clinic of Internal Medicine I, Jena University Hospital, 07747 Jena, Germany; 5 Klinik für Kinder und Jugendmedizin, Universitätsklinikum Jena, Am Klinikum 1, 07747 Jena, Germany

**Keywords:** Autophagy, Cell cycle, Cell sorting, Cyto-ID, DRAQ5, Metabolic analysis

## Abstract

**Background:**

Autophagy plays an essential role in maintaining cellular homeostasis and in the response to cellular stress. Autophagy is also involved in cell cycle progression, yet the relationship between these processes is not clearly defined.

**Results:**

In exploring this relationship, we observed that the inhibition of autophagy impaired the G2/M phase-arresting activity of etoposide but enhanced the G1 phase-arresting activity of palbociclib. We further investigated the connection of basal autophagy and cell cycle by utilizing the autophagosome tracer dye Cyto-ID in two ways. First, we established a double-labeling flow-cytometric procedure with Cyto-ID and the DNA probe DRAQ5, permitting the cell cycle phase-specific determination of autophagy in live cells. This approach demonstrated that different cell cycle phases were associated with different autophagy levels: G1-phase cells had the lowest level, and G2/M-phase cells had the highest one. Second, we developed a flow-cytometric cell-sorting procedure based on Cyto-ID that separates cell populations into fractions with low, medium, and high autophagy. Cell cycle analysis of Cyto-ID-sorted cells confirmed that the high-autophagy fraction contained a much higher percentage of G2/M-phase cells than the low-autophagy fraction. In addition, Cyto-ID-based cell sorting also proved to be useful for assessing other autophagy-related processes: extracellular flux analysis revealed metabolic differences between the cell populations, with higher autophagy being associated with higher respiration, higher mitochondrial ATP production, and higher glycolysis.

**Conclusion:**

This work provides clear evidence of high autophagy in G2/M-phase cells by establishing a novel cell sorting technique based on Cyto-ID.

**Supplementary Information:**

The online version contains supplementary material available at 10.1186/s11658-022-00334-8.

## Introduction

Macroautophagy (henceforth autophagy) is a cytoprotective mechanism critically involved in physiological and pathophysiological processes [1–3]. It is a constitutive activity for bulk degradation of damaged, aged, or surplus organelles and aberrant protein aggregates, thus preserving cellular homeostasis. It also serves as an inducible response to stress insults, allowing cells to cope with, e.g., starvation, hypoxia, or oxidative damage. The autophagic pathway comprises the sequestration of cytoplasmic constituents through the formation of double-membrane autophagosomes that eventually fuse with lysosomes to create autolysosomes [1, 4]. The latter degrade their cargo, thereby not only removing potentially harmful elements but also facilitating the reutilization of building blocks, e.g., for protein synthesis, gluconeogenesis, and energy production. At the molecular level, autophagy is regulated by the coordinated action of autophagy-related (ATG) genes [1, 4]. Autophagy is implicated in many human diseases, and its therapeutic targeting has gained traction in recent years, particularly for the treatment of cancer [1–8].

Autophagy, therefore, is a fundamental activity in eukaryotic cells, and it is hence hardly surprising that it is also involved in cell cycle progression and regulation [9–12]. Autophagy and cell cycle are linked by a variety of mechanisms that are governed by common signaling pathways. Particular mention here deserves mTORC1 signaling, which plays a crucial role in controlling both autophagy and cell growth [13, 14]: mTORC1 promotes proliferation and suppresses autophagy, pointing to an inverse relationship between these cellular processes [9–12]. This concept is further reinforced by observations that cell-cycle-promoting protein kinases, such as cyclin-dependent kinases (CDKs), prevailingly impinge on the autophagic pathway [15]. Vice versa, both endogenous and pharmacological inhibitors of CDKs have been reported to increase autophagy [16–18]. Autophagy and cell cycle are additionally interconnected through the DNA damage response that entails the suspension of cell cycle progression as well as the stimulation of autophagy; the former allows time for proper DNA repair, while the latter supports the DNA repair machinery [19–22]. DNA damage-mediated autophagy and cell cycle responses are prominently intertwined by the tumor suppressor protein p53 [23, 24].

A possible interdependence of cell cycle phase and autophagy has been addressed in several studies. While the findings widely agree that autophagy is differentially regulated during the cell cycle, they diverge as to which cell cycle phase exhibits highest autophagic activity. The majority of reports demonstrated a link of higher autophagy levels to interphase [25–27], and autophagy inducers as diverse as starvation, the mTORC1 inhibitor sirolimus (also known as rapamycin), ABT-737, tunicamycin, and lithium-elicited autophagy preferentially in the G1 and S phases of the cell cycle [28]. Likewise, G1 cell cycle arrest mediated by drugs such as celecoxib, metformin, magnolin, and dimethyl fumarate was found to be associated with elevated autophagy [29–32]. Yet some papers have reported highly active autophagy in mitosis [33–36], and others have related autophagy activation to a halt of the cell cycle in G2 or mitosis. Concurrent induction of autophagy and G2/M cell cycle arrest was observed in response to agents such as plumbagin, resveratrol, artesunate, and a boswellic acid analog [37–40] as well as after ionizing radiation [41, 42]. In addition, removal of damaged mitochondria by autophagy can be associated with cell cycle arrest in G2/M [43]. By contrast, the notion of a coordinated interplay of autophagy and cell cycle was challenged by a study showing that starvation- and sirolimus-induced accumulation of autophagosomes occurred at all stages of the cell cycle [44].

All told, the relation between autophagy and cell cycle is far from being resolved, and further work is required to understand their interconnection. We, therefore, set out to re-examine the interaction of autophagy and cell cycle at its basis. To this end, we took advantage of Cyto-ID, a fluorescent dye for selective staining of autophagosomes [45–47], in two ways: First, we conducted double-labeling flow cytometry with Cyto-ID and the live-cell-permeant DNA marker DRAQ5, allowing for simultaneous monitoring of autophagy and cell cycle phases. Second, we developed a Cyto-ID-based flow-cytometric cell sorting procedure that separates cell populations into subgroups with low, medium, and high autophagy suited for downstream culturing of separated cells. Our analyses revealed a clear association between differences in constitutive autophagy and cell cycle phase—cells with low autophagy systematically predominated in the G1 phase and cells with high autophagy in the G2/M phase.

## Materials and methods

### Cell culture

Jurkat and MOLM-13 cells were purchased from the DSMZ (Braunschweig, Germany). Cells were cultured in RPMI 1640 medium with stable l-glutamine (Lonza, Cologne, Germany) supplemented with 10% (Jurkat) or 20% (MOLM-13) FCS (Capricorn Scientific, Ebsdorfergrund, Germany), 100 units/ml penicillin G sodium salt, and 100 µg/ml streptomycin sulfate (Lonza). Cells were maintained in a humidified atmosphere at 37 °C and 5% CO_2_. Cells were tested to be negative for mycoplasma with the qPCR Mycoplasma Test Kit from Applichem (Darmstadt, Germany).

### Treatment of cells

Cells were seeded in 12-well tissue culture plates at 150,000 (Jurkat) or 200,000 (MOLM-13) cells per well. Cells were exposed to 0.1–0.5 µM etoposide (provided by the Jena University Hospital Pharmacy) for 6–48 h or to 25–400 nM palbociclib (MedChemExpress, Monmouth Junction, NJ, USA) for 48 h to induce autophagy. Cells were exposed to 2 mM 3-methyladenine (3-MA; Biomol, Hamburg, Germany) for 24 h or 48 h, or to 10 or 25 µM chloroquine (CQ; Enzo Life Sciences, Lörrach, Germany) for 1–48 h to inhibit autophagy.

### Flow-cytometric analysis of cell death

Cell death was determined by propidium iodide (PI) uptake analysis. After harvesting, cells were incubated in 2 µg/ml PI (Sigma-Aldrich, Deisenhofen, Germany) in PBS at 4 °C immediately before analysis. In total, 10,000 cells per sample were analyzed on a BD (Heidelberg, Germany) FACSCanto II using BD FACSDiva software. Data were gated on the basis of forward light scatter area (FSC-A) versus sideward light scatter area (SSC-A) to exclude debris.

### Flow-cytometric analysis of autophagy

Cells were stained with Cyto-ID (Enzo Life Sciences) according to the manufacturer’s recommendations. In brief, cells were washed with indicator-free medium (IFM), consisting of phenol-red-free RPMI 1640 medium (Capricorn Scientific) containing 5% FCS and 2 mM l-glutamine (Lonza), and incubated in Cyto-ID at a dilution of 1:1000 in IFM for 30 min at 37 °C. After washing and resuspension in IFM, PI was added to a final concentration of 2 µg/ml PI immediately before analysis. 10,000 cells per sample were analyzed on a FACSCanto II using FACSDiva software. Data were gated based on FSC-A versus SSC-A to exclude debris and further gated on PI-negative populations to exclude dead cells.

### Flow-cytometric analysis of DNA content

Protocol I, analysis of PI-stained ethanol-fixed cells: Cells fixed in 70% ethanol at − 20 °C for at least 2 h were washed and resuspended in PBS containing 1% glucose, 50 µg/ml RNase A (Sigma-Aldrich) and 50 µg/ml PI, and incubated under light exclusion for 45 min at 4 °C. Protocol II, analysis of DRAQ5-stained live cells, applied only in Cyto-ID/DRAQ5 double-labeling analysis: Cells were adjusted to a density of 400,000 cells/ml and incubated with 10 µM DRAQ5 (Thermo Fisher Scientific, Dreieich, Germany) in IFM for 15 min at 0 °C (i.e., DRAQ5 staining was carried out subsequent to Cyto-ID staining). In both methods, 20,000 cells per sample were analyzed on a FACSCanto II. Data were gated based on FSC-A versus SSC-A to exclude debris and on FSC-A versus FSC width (FSC-W) to exclude aggregates. The different cell cycle phases were quantified using FACSDiva software; cells with sub-G1 DNA content were included in some Protocol I analyses.

### Flow-cytometric cell sorting on the basis of Cyto-ID staining

1 × 10^7^ cells were stained with Cyto-ID as described, resuspended in 1 ml IFM, filtered through 35-µm mesh and incubated with 1 µM Sytox Blue (Thermo Fisher Scientific) immediately before sorting. Cells were sorted into three subpopulations of approximately equal number according to the Cyto-ID fluorescence intensity, i.e., into populations with low, medium and high Cyto-ID fluorescence. To minimize the starvation period during the sorting procedure, the collection tubes were prefilled with 1 ml of complete growth medium or IFM, depending on the subsequent analysis. Sorting was done on a BD FACSAria Fusion at 45 psi using an 85 µM nozzle at 4 °C. Debris and aggregates were excluded from the sorting using a sequential gating strategy relying on FSC-A versus SSC-A followed by FSC height (FSC-H) versus FSC-W and SSC height (SSC-H) versus SSC width (SSC-W). Dead cells were excluded by gating on Sytox Blue-negative cells. The dot plots shown in Additional file 4: Fig. S4 were created with FlowJo version 10.5.0 (TreeStar, Ashland, OR, USA). For downstream culturing, cells were seeded in 12-well tissue culture plates at 100,000 cells/well.

### Flow-cytometric cell sorting on the basis of DRAQ5 staining

2 × 10^6^ cells were stained with DRAQ5 as described, resuspended in 1 ml IFM, filtered through 35-µm mesh and incubated with 1 µM Sytox Blue immediately before sorting. Cells were sorted into G1, S and G2/M phase fractions. Sorting was done on a BD FACSAria Fusion at 45 psi using an 85 µM nozzle at 4 °C. Debris and aggregates were excluded from the sorting using a sequential gating strategy relying on FSC-A versus SSC-A followed by FSC-H versus FSC-W and SSC-H versus SSC-W. Dead cells were excluded by gating on Sytox Blue-negative cells. Cells were subjected to analyses immediately after sorting since DRAQ5's genotoxicity precludes culturing of DRAQ5-exposed cells [48].

### Real-time RT-PCR

Total RNA was isolated using the Peqgold Total RNA Kit including DNase digestion (Peqlab, Erlangen, Germany). RNA was transcribed into cDNA using the Omniscript RT Kit (Qiagen, Hilden, Germany). Real-time PCR was conducted on a Thermo Fisher Scientific Applied Biosystems 7900HT Real-Time PCR system. Target gene expression levels were normalized to *B2M* expression levels. Reactions were done in duplicate using Applied Biosystems Gene Expression Assays (*ULK1*: Hs00177504_m1, *MAP1LC3B*: Hs00797944_s1, *CCNB1*: Hs01030099_m1, *PLK1*: Hs00983227_m1, *B2M*: Hs00187842_m1) and Universal PCR Master Mix. All procedures were conducted as per the manufacturers' instructions. The relative gene expressions were calculated by the 2(^−ΔΔCt^) method.

### Immunoblotting

Lysates were prepared either immediately after sorting or after 1-h cultivation of sorted cells. 350,000 cells of each fraction were centrifuged at 250×*g* for 5 min and resuspended in 35 µl RIPA buffer [50 mM Tris/HCl (pH 8.0), 150 mM NaCl, 1 mM EDTA, 1% Triton X-100, 1% sodium deoxycholate and 0.1% SDS] supplemented with protease and phosphatase inhibitor cocktails (Sigma-Aldrich), followed by brief sonication (5 cycles with 30 s on and 30 s off with high intensity at 4 °C) in a Bioruptor Plus (Diagenode, Seraing, Belgium) sonication device. All samples were diluted 1:6 in 6 × SDS-sample buffer (35% β-mercaptoethanol, 350 mM Tris/HCl pH 6.8, 30% glycerol, 10% SDS, 0.25% bromophenol blue) and heated at 95 °C for 7 min. 10–20 µl sample volume per lane were separated by standard SDS-PAGE on 7–12–15% three-step gels and electrophoretically transferred onto PVDF membranes (Bio-Rad, Munich, Germany). After blocking in TBS (pH 7.25) containing 5% dry milk and 0.05% Tween-20, the membranes were incubated with primary antibodies overnight at 4 °C. Antibodies used: LC3B (1:1000; Cell Signaling Technologies, Frankfurt/Main, Germany, 2775S), Cyclin B1 (1:1000; BD, 554176) and pS10-H3 (1:1000; Merck Millipore, Darmstadt, Germany, 06-570). Equal loading of protein was verified by using β-actin antibodies (1:10,000; Sigma-Aldrich, A5441). Peroxidase-conjugated anti-mouse (1:10,000; 5220-0341) or anti-rabbit IgG (1:5000; Seracare, Milford, MA, USA, 5220-0336) was used as secondary antibodies followed by detection of specific signals using Pierce ECL Western Blotting substrate (Thermo Fisher Scientific) and WesternBright Sirius HRP substrate (Advansta, San Jose, CA, USA) on an Amersham Imager 600 (GE Healthcare, Munich, Germany).

### Metabolic analysis

Cell Mito Stress Tests were done in sextuplicates using a Seahorse XFe96 Extracellular Flux Analyzer (Agilent Technologies, Waldbronn, Germany). Following cell sorting, cells were switched to Seahorse XF RPMI medium (pH preadjusted to 7.4) supplemented with 10 mM glucose, 2 mM L-glutamine (Sigma-Aldrich), 1 mM sodium pyruvate and 10% fetal calf serum (FCS; Thermo Fisher Scientific). Cells were plated at 70,000 cells per well in Seahorse XF96 cell culture microplates and left to equilibrate in a CO_2_-free incubator at 37 °C for 1 h. Oxygen consumption rate (OCR) and extracellular acidification rate (ECAR) were determined according to Seahorse protocols with some modifications. In brief, OCR (pmol/min) and ECAR (mpH/min) were measured three times each at baseline and after sequential injections of 2 µM oligomycin (Abcam, Berlin, Germany), 15 µM 2,4-dinitrophenol, and 2 µM antimycin A (Sigma-Aldrich) in cycles of 3 min mixing and 3 min measuring. The datasets were analyzed with Wave software (Agilent Technologies). ATP production was calculated by subtracting the average of the three OCR values after oligomycin injection from the average of the three OCR values before oligomycin injection.

### Statistical analysis

Statistical significance of differences between experimental groups was determined using paired two-tailed Student’s *t* test. The threshold of significance was defined as *P* < 0.05 without further differentiation. Statistical difference was assessed only where relevant; the lack of an asterisk or a hash mark thus does not imply lack of significant difference.

## Results

### The autophagy inhibitor 3-MA prevents etoposide-mediated cell cycle effects

**Fig. 1:**
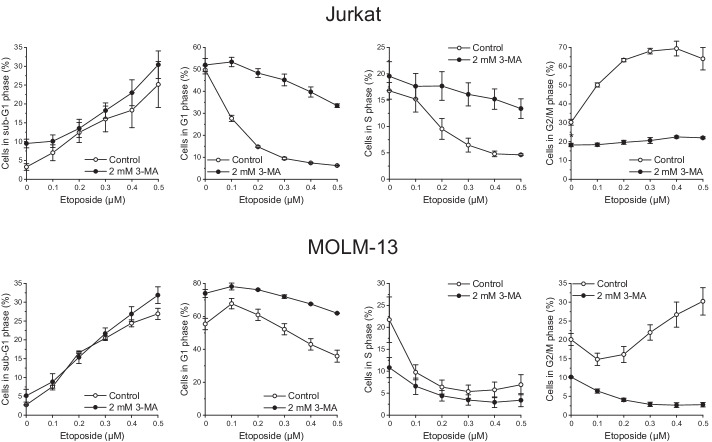
Autophagy inhibition affects etoposide-induced cell cycle arrest. Cells were exposed to etoposide with or without 3-MA for 48 h. Cell cycle phases were determined by flow-cytometric analysis of PI-stained ethanol-fixed cells. Mean ± SEM of three independent measurements is shown

This study aimed at shedding light on the interaction of autophagy and cell cycle progression. To begin, we applied etoposide [49], a topoisomerase II inhibitor that induces both G2/M cell cycle arrest [50, 51] and autophagy [52–54]. We employed suspension cells to obviate the necessity of enzyme treatment for cell harvest because trypsin treatment can provoke considerable cell physiological alterations [55] potentially also interfering with autophagy. We used Jurkat and MOLM-13 leukemia cells, cell lines with different p53 status, a feature that may impact the cell cycle and the autophagy response [23, 24]; Jurkat cells have mutant p53, and MOLM-13 cells have wild-type p53 [56, 57]. We exposed the cells to etoposide alone and in conjunction with 3-MA, one of the most commonly used autophagy inhibitors [58], and performed cell cycle analysis of PI-stained ethanol-fixed cells (Fig. 1). Etoposide alone caused a concentration-dependent accumulation of Jurkat cells in the G2/M phase and a biphasic response in MOLM-13 cells; in the latter, increasing concentrations of etoposide produced first an accumulation of cells in the G1 phase and then in the G2/M phase. These effects were thwarted by 3-MA, providing initial evidence of an interrelationship between autophagy and cell cycle.

### G2/M-phase cells have higher autophagy than G1-phase cells

**Fig. 2:**
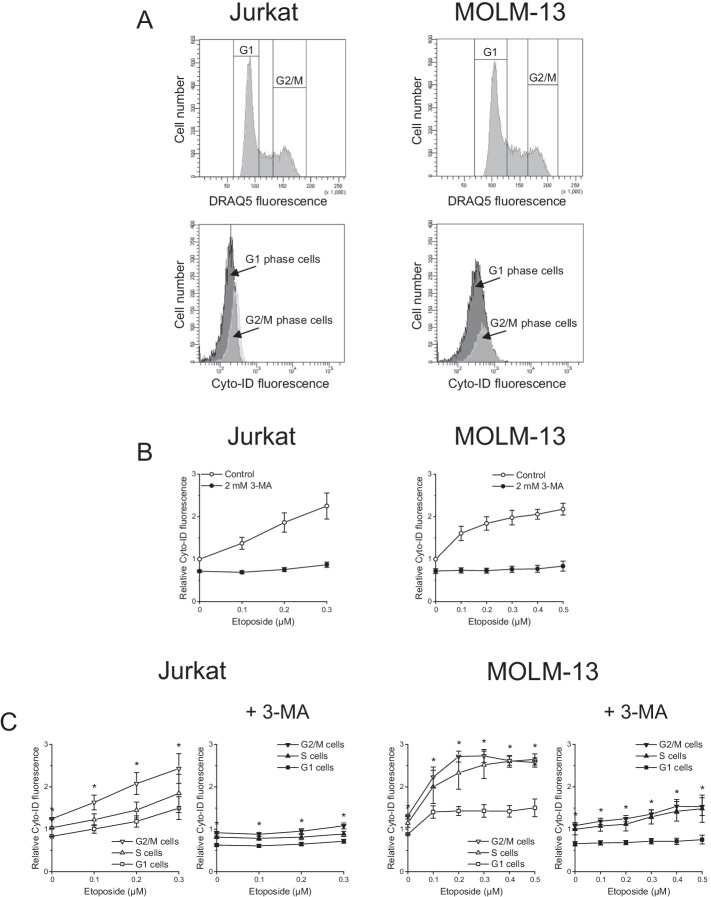
G2/M-phase cells have higher autophagy than G1-phase cells. Autophagy and cell cycle phases were determined by flow-cytometric analysis of Cyto-ID + DRAQ5 double-stained cells. **A** Representative histograms of untreated cells. **B, C** Cells were exposed to etoposide with or without 3-MA for 24 h (Jurkat) or 48 h (MOLM-13). Cyto-ID fluorescence intensities were normalized to the mean Cyto-ID fluorescence intensities of untreated cells. Mean ± SEM of three independent measurements is shown (G1 cells versus G2/M cells: *P < 0.05)

We replicated these analyses in live cells using the supravital DNA dye DRAQ5 and obtained similar results (Additional file 1: Fig. S1). Jurkat cells were exposed to etoposide for only 24 h since a considerable increase in autophagy became manifest already after this period (compare Fig. 4A), while MOLM-13 cells were treated for 48 h as in the PI-staining protocol. To simultaneously assess cell cycle distribution and autophagy, we costained cells with DRAQ5 and Cyto-ID, thus enabling the cell-cycle-phase-specific monitoring of autophagy (Fig. 2A). Figure 2B shows that etoposide induced a concentration-dependent increase in Cyto-ID fluorescence indicative of autophagy activation. 3-MA predictably blocked the Cyto-ID fluorescence increase.

As with LC3 immunoblotting for the detection of autophagy [58], Cyto-ID fluorescence alone is insufficient for the estimation of autophagic flux. Yet the use of agents that prevent autophagosome turnover, such as CQ, can provide evidence of alterations in autophagic flux; an additive or supra-additive effect of combined treatment with the putative autophagy inducer and the autophagosome turnover blocker is indicative of enhanced autophagic flux [58]. Accordingly, we compared etoposide alone versus etoposide plus CQ. The combination treatment resulted in a supra-additive effect relative to treatment with etoposide or CQ alone in both cell lines (Additional file 2: Fig. S2).

Cyto-ID/DRAQ5 double staining also revealed that in untreated cells, i.e., cells with basal autophagy, different cell cycle phases were associated with different autophagy levels: the highest level was found in G2/M-phase cells and the lowest in G1-phase cells (Fig. 2C). This difference was also evident, at a lower level, in 3-MA-treated cells. Furthermore, treatment with etoposide led to autophagy activation in all cell cycle phases, though strongest activation arose in G2/M-phase cells. The effects occurred in both p53 wild-type and mutant cells, indicating that p53 was not critically involved.

**Fig. 3:**
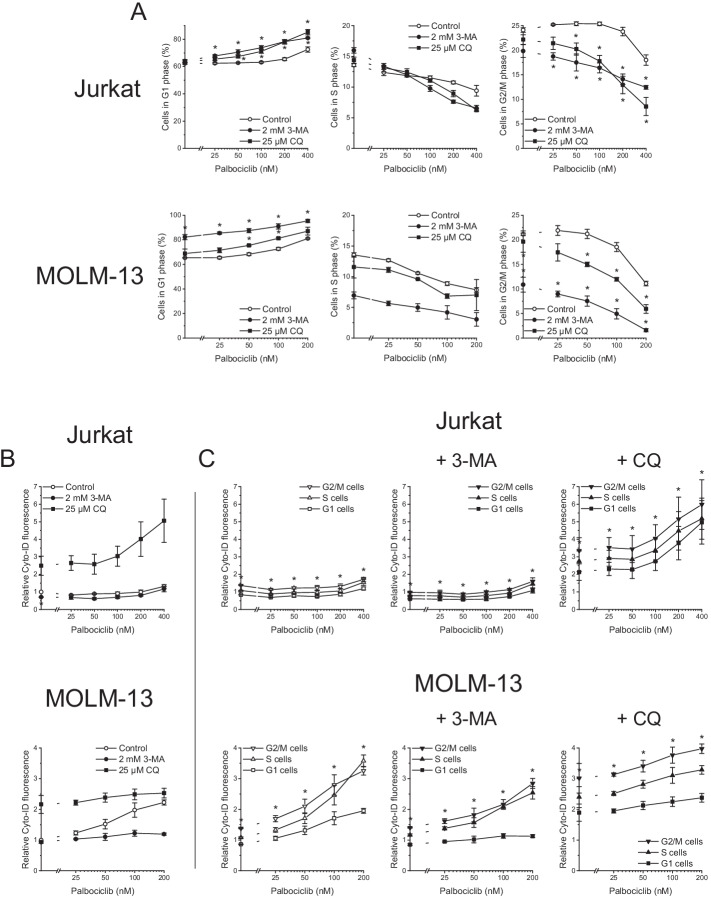
Autophagy inhibition enhances palbociclib-induced G1-phase arrest. Cells were exposed to palbociclib with or without autophagy inhibitors for 48 h. Autophagy and cell cycle phases were determined by flow-cytometric analysis of Cyto-ID + DRAQ5 double-stained cells. **A** Effect of palbociclib on cell cycle distribution. **B, C** Effect of palbociclib on autophagy. Cyto-ID fluorescence intensities were normalized to the mean Cyto-ID fluorescence intensities of untreated cells. Mean ± SEM of three independent measurements is shown (A: 3-MA or CQ versus control, C: G1 cells versus G2/M cells: *P < 0.05)

To complement these data with the evaluation of a G1-phase blocker, we conducted similar analyses using the CDK4/6 inhibitor palbociclib, which induces G1 cell cycle arrest [59] along with autophagy [17, 18]. Palbociclib treatment predictably increased the fraction of Jurkat and MOLM-13 cells in the G1 phase (Fig. 3A). Coexposure to the autophagy inhibitors 3-MA or CQ further increased the percentage of G1-phase cells, thus enhancing palbociclib’s cell-cycle-arresting activity. Palbociclib also evoked a rise in Cyto-ID fluorescence. This, however, became evident in Jurkat cells only upon cotreatment with CQ (Fig. 3B). In any case, the G2/M-phase cells again showed the strongest Cyto-ID fluorescence throughout the measurements (Fig. 3C).

**Fig. 4:**
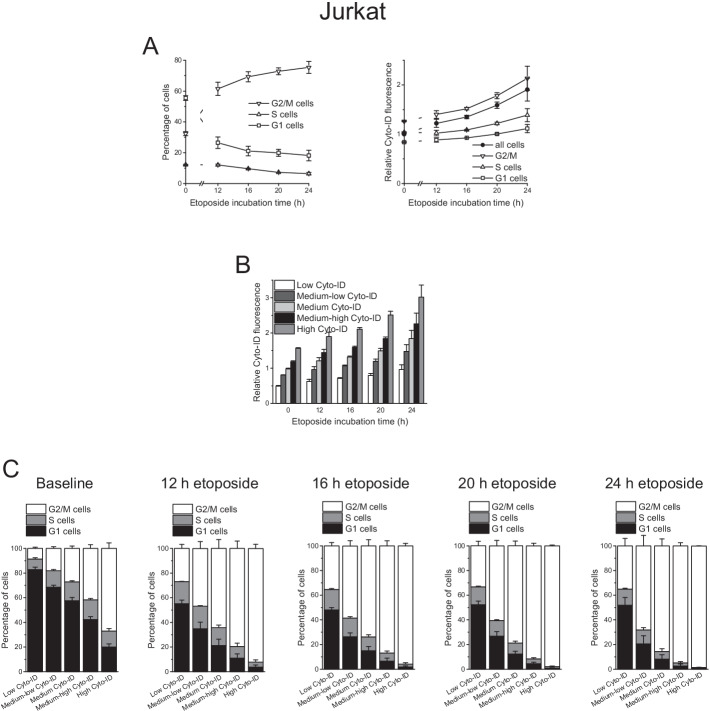
Cell cycle phase and autophagy are tightly associated. Cells were exposed to 0.2 µM etoposide for the indicated times. Autophagy and cell cycle phases were determined by flow-cytometric analysis of Cyto-ID + DRAQ5 double-stained cells. **A** Effect of etoposide on cell cycle phase distribution and autophagy. **B, C** Cell populations were grouped into quintiles based on their Cyto-ID fluorescence intensities (Fig. S3A). **B** Relative Cyto-ID fluorescence intensities of the quintiles of cells. Cyto-ID fluorescence intensities were normalized to the mean Cyto-ID fluorescence intensity of untreated cells. **C** Cell cycle phase distribution in the quintiles of cells. Mean ± SEM of two (12-h and 16-h treatment) or three independent measurements is shown

To gain additional insight into the interplay of autophagy and cell cycle phase, we assessed the effect of etoposide over a time course of 12–24 h in Jurkat cells (Fig. 4A). For a close analysis of the data, we grouped the cell populations into quintiles according to their Cyto-ID fluorescence at each timepoint (Fig. 4B and Additional file 3: Figure S3A). We generated cell cycle profiles of each quintile of cells, demonstrating that autophagy level and cell cycle phase were unambiguously associated—the higher the autophagy level, the more cells in the G2/M phase (Additional file 3: Fig. S3B presents the cell cycle histograms at baseline, i.e., for untreated cells with basal autophagy, and after 24-h exposure to 0.2 µM etoposide, and Fig. 4C presents the quantifications for all conditions). For example, at baseline, of cells with lowest autophagy, 82.8 ± 2.02% were in G1 and 8.5 ± 1.00% in G2/M phase, while of cells with highest autophagy, 19.9 ± 2.58% were in G1 and 67.0 ± 4.49% in G2/M phase.

### Cyto-ID-based cell sorting

So far, our findings had clearly pointed to particularly high autophagy in G2/M-phase cells. To test this further, we established a flow-cytometric cell-sorting method for the separation of cells with low, medium, and high basal autophagy (henceforth referred to as Aut^LO^, Aut^ME^, and Aut^HI^, respectively) on the basis of Cyto-ID fluorescence (the gating strategy for Cyto-ID-based cell sorting is shown Additional file 4: Fig. S4). We beforehand checked critical parameters for Cyto-ID-based cell sorting: Cyto-ID was not cytotoxic over a sustained incubation of 4 h (Additional file 5: Fig. S5A); at 4 °C, the Cyto-ID fluorescence did not change over the measurement period of 90 min, whereas it rapidly declined at room temperature and even more so at 37 °C (Additional file 5: Fig. S5B); the sorting procedure per se had no effect on the cells’ autophagy (Additional file 5: Fig. S5C). We also checked the possibility of a mere association of Cyto-ID fluorescence intensity with cell size and detected only minute differences in size between cells with low, medium, and high Cyto-ID fluorescence (Additional file 5: Fig. S5D), thus excluding the possibility of higher Cyto-ID fluorescence simply being due to potentially higher autophagosome numbers in bigger cells.

**Fig. 5:**
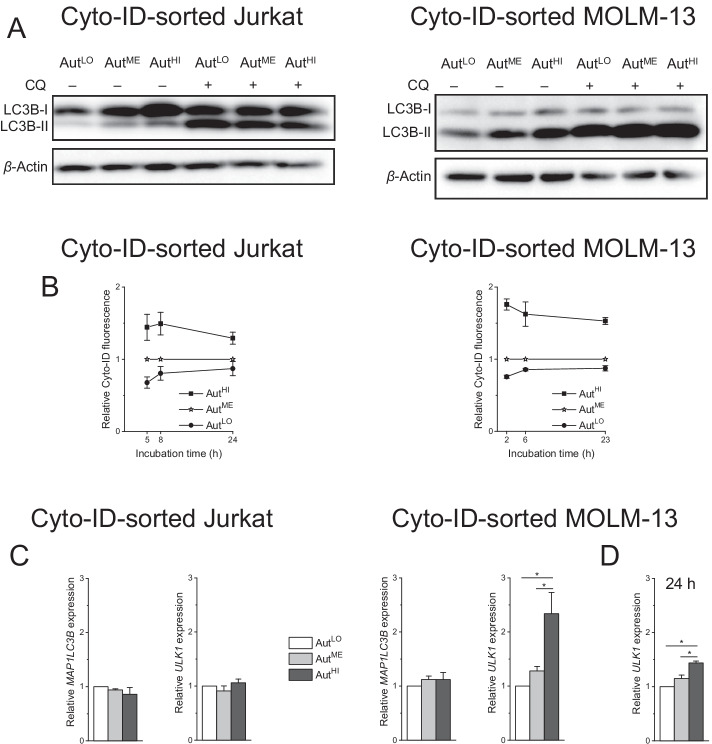
Cyto-ID-sorted cells display different autophagy levels over at least 24 h. Cells were flow-cytometrically sorted on the basis of their Cyto-ID fluorescence intensity into subpopulations with low, medium, and high Cyto-ID fluorescence (Aut^LO^, Aut^ME^ and Aut^HI^, respectively). **A** Representative immunoblots of lysates from sorted cells. Lysates were prepared after 1-h cultivation of sorted cells in the absence or presence of 10 µM CQ. **B** Sorted cells were incubated for the indicated times, and autophagy was determined by flow-cytometric analysis of Cyto-ID-stained cells. Cyto-ID fluorescence intensities of the three fractions were normalized to the Cyto-ID fluorescence intensity of Aut^ME^. **C, D** RNA was prepared either approximately 1 h after sorting (**C**) or after 24-h cultivation of sorted cells (**D**). mRNA expression levels were determined by real-time RT-PCR and normalized to *B2M* expression levels. Mean ± SEM of three (**C**–**D**) or four (**B**) independent measurements is shown (*P < 0.05)

LC3B immunoblots confirmed the distinct autophagy levels of the three fractions (Fig. 5A). Upon assessing the stability of the different autophagy levels of the three populations, we found that differences persisted for at least 24 h after sorting, although they tended to converge over time (Fig. 5B). As a first step toward a differential characterization of Aut^LO^, Aut^ME^, and Aut^HI^, we conducted gene expression analyses. Although autophagy is basically a cytoplasmic pathway, it can also be subject to transcriptional regulation [60–62]. To examine whether variations in basal autophagy were reflected by differences in the expression of ATG genes, we determined the relative mRNA abundance of two ATG genes that have particularly often shown transcriptional regulation, the LC3B-coding gene *MAP1LC3B* and *ULK1* [61]. While the former was equally expressed in the three populations of both cell lines, the expression level of the latter displayed an association with the level of autophagy in MOLM-13 cells, i.e., *ULK1* was expressed in the order Aut^HI^ > Aut^ME^ > Aut^LO^ (Fig. 5C). Remarkably, differences in *ULK1* expression were still evident 24 h after sorting, although to a reduced degree (Fig. 5D). It should be noted, however, that *ULK1* was uniformly expressed in the three fractions of Jurkat cells, demonstrating that autophagy variability was not strictly associated with altered *ULK1* expression.

### Cells with high autophagy are more metabolically active than cells with low autophagy

**Fig. 6:**
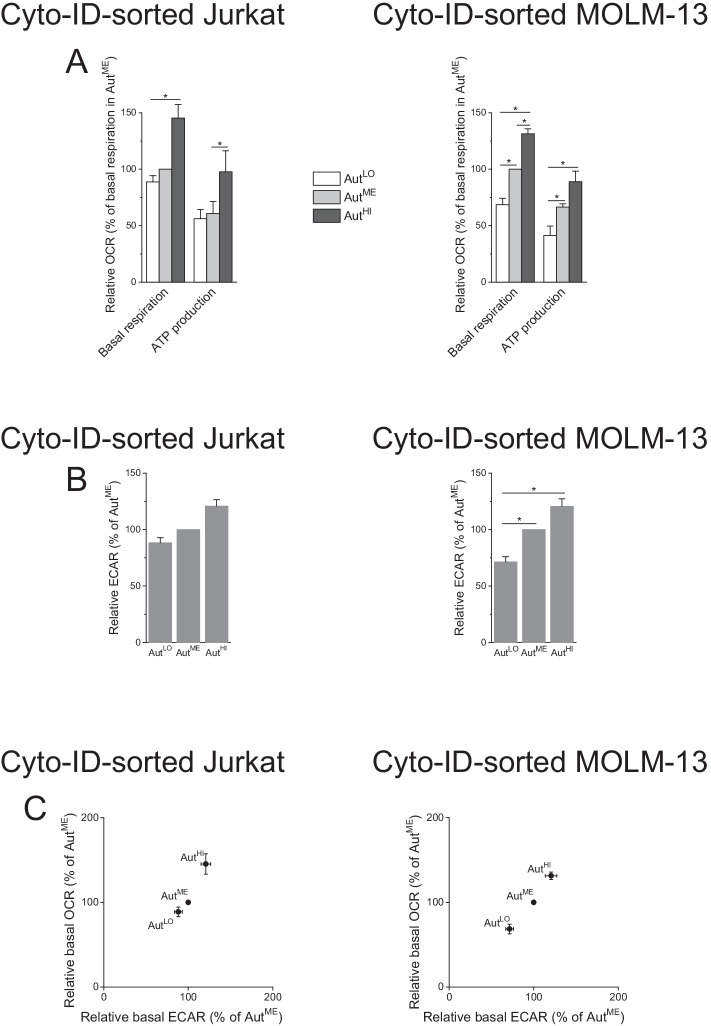
Autophagic and metabolic activities are associated. Cells were flow-cytometrically sorted on the basis of their Cyto-ID fluorescence intensity into subpopulations with low, medium, and high Cyto-ID fluorescence (Aut^LO^, Aut^ME^, and Aut^HI^, respectively). The subpopulations were subjected to metabolic analysis immediately after sorting. **A** OCR as a proxy for oxidative metabolism was measured with a Seahorse XFe96 Analyzer using a Cell Mito Stress Test. ATP production was calculated from OCR data (see Fig. S6 for details). **B** ECAR was measured as a proxy for glycolytic activity. OCR and ECAR of the three fractions were normalized to OCR and ECAR of Aut^ME^. **C** Energetic maps of sorted cells generated from OCR and ECAR data presented in A and B, respectively. Means ± SEM of each three independent measurements are shown; each biological replicate consisted of six readings (*P < 0.05)

Autophagy and cellular metabolism are intimately linked [63–65]. So, to further characterize the Cyto-ID-sorted cell populations, we performed metabolic measurements with a Seahorse XFe96 Analyzer [66]. Using the Seahorse Cell Mito Stress Test, we measured key mitochondrial functions by determining the OCR of cells utilizing sequential reagent injections (Additional file 6: Fig. S6). We recorded higher basal respiration and higher ATP production in Aut^HI^ compared with Aut^ME^ and Aut^LO^ in both cell lines (Fig. 6A). In parallel, we measured the ECAR to assess the glycolytic production of lactate/H^+^, revealing that baseline glycolysis was also highest in Aut^HI^ (Fig. 6B). Mapping the OCR of basal respiration versus glycolysis-related ECAR illustrates that higher autophagic activity was strictly associated with higher bioenergetic activity in the two cell lines (Fig. 6C).

### Cells with high autophagy are preferentially in the G2/M phase

**Fig. 7:**
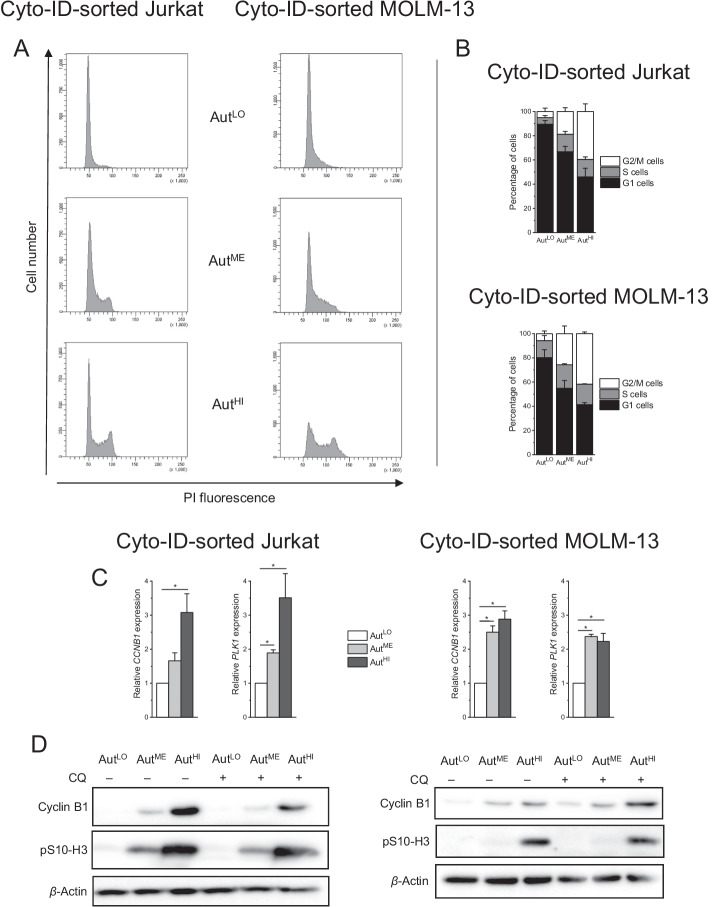
Cells with high autophagy are preferentially in the G2/M phase of the cell cycle. Cells were flow-cytometrically sorted on the basis of their Cyto-ID fluorescence intensity into subpopulations with low, medium, and high Cyto-ID fluorescence (Aut^LO^, Aut^ME^, and Aut^HI^, respectively). **A, B** Cells were fixed in ethanol approximately 1 h after sorting. Cell cycle phases were determined by flow-cytometric analysis of PI-stained ethanol-fixed cells. **A** Representative histograms of cell populations. **B** Quantification of cell cycle phases. **C** RNA was prepared approximately 1 h after sorting. mRNA expression levels were determined by real-time RT-PCR and normalized to *B2M* expression levels. Mean ± SEM of three independent measurements is shown (*P < 0.05). **D** Representative immunoblots of lysates from sorted cells. Lysates were prepared after 1-h cultivation of sorted cells in the absence or presence of 10 µM CQ. The loading controls are the same as in Fig. 5A since LC3B, cyclin B1, pS10-H3, and β-actin were detected on the same blots

To return to our primary purpose, the elucidation of the relationship between autophagy and cell cycle, we subjected the Cyto-ID-sorted cell populations to cell cycle analyses. Our findings matched those shown in Fig. 4: less than 10% of Aut^LO^ but about 40% of Aut^HI^ were found in the G2/M phase in both Jurkat and MOLM-13 cells (Fig. 7A, B). We attempted to further substantiate the evidence for this interaction by real-time RT-PCR and immunoblot analysis of cell cycle regulated genes. *CCNB1* (encoding cyclin B1) and *PLK1* were used as marker genes and cyclin B1 and phosphohistone (Ser10) H3 (pS10-H3) were used as marker proteins of cells in the G2/M phase of the cell cycle [67]. Figure 7C, D shows that G2/M marker abundance was systematically increased in Aut^HI^.

We also adopted a reciprocal approach to address the interrelation between autophagy and cell cycle by sorting of cells into G1, S, and G2/M fractions on the basis of their DRAQ5 signal followed by Cyto-ID staining. Again, the G1 phase was associated with lowest autophagy, and vice versa, the G2/M phase was associated with highest autophagy (Additional file 7: Fig. S7).

## Discussion

Here we explored the relationship between basal autophagy and cell cycle in leukemia cells. Importantly, we showed that the G2/M phase was linked to highest autophagy. The evidence for this conclusion came from two experimental approaches: the simultaneous monitoring of autophagy and cell cycle, and the sorting of cells into populations with distinct autophagy levels.

As our first evidence for the interdependence of autophagy and cell cycle, however, we observed that the autophagy inhibitor 3-MA abrogated the etoposide-induced accumulation of G2/M-phase cells. This observation is in line with studies reporting that 3-MA or CQ prevented other compounds from eliciting cell cycle arrest [31, 38–40]. More importantly, the exposure to 3-MA alone increased the proportion of cells in the G1 phase (Fig. 1 and Additional file 1: Fig. S1 at 0 µM etoposide), indicative of a G1 cell cycle block. This result suggests that a certain minimum autophagic activity is essential for cell cycle progression. A similar conclusion was reached upon investigating ULK1/ATG13 double-knockout cells [36]. Our examination of the effects of palbociclib additionally supports this conclusion, since it showed that, although the induction of G1-phase arrest was accompanied by increased autophagy, preventing the latter further fueled the former. An accessory implication of this finding with potential clinical relevance is that the inhibition of autophagy might enhance the cytostatic action of CDK4/6 inhibitors [59].

To deepen our understanding of the interrelation between basal autophagy and cell cycle, we first established a cell-cycle-phase-specific delineation of autophagy by Cyto-ID/DRAQ5 double-staining analysis. This approach demonstrated strongest Cyto-ID fluorescence in G2/M-phase cells. This observation is in keeping with a study that detected increased levels of LC3B in G2/M-phase cells by flow cytometric analysis of immunofluorescent-labeled LC3B [68]. Our results were substantially the same in both p53 mutant Jurkat and p53 wild-type MOLM-13 cells, indicating that p53 did not play a major role here, while in a study on colon cancer cells, p53 was shown to affect autophagy in a cell-cycle-dependent manner [69]. In exploring the functional consequences of cell-to-cell differences in basal autophagy, Gump et al. found that the stochastic variability in autophagic activity determined the apoptotic response to death ligands [70]. Our data show that the range of cell-to-cell differences in autophagy was narrower in the individual cell cycle phases than in the total cell population, suggesting that the cell-population-intrinsic heterogeneity in autophagy is in part accounted for by the cell cycle phase.

We then established a flow cytometry method for the separation of cells based on their differences in basal autophagy. We are aware of only one previous approach for autophagy-based sorting of cells: Gump et al. employed cells constitutively expressing a tandem labeled fluorescent reporter (mCherry-EGFP-LC3B), where a high red/green fluorescence ratio indicates cells with enhanced autophagic flux [70, 71]. We used Cyto-ID staining that, though not directly measuring autophagic flux, has two advantages. First, cells do not undergo any manipulation other than Cyto-ID staining prior to sorting. The method thus avoids the potential pitfalls arising from genetic manipulations in general, which can introduce genetic variation even when considered to be neutral [72], and in particular those caused by ectopic expression of chimeric GFP-LC3B [58]. Second, since our procedure does not involve the generation of stable reporter cell lines, it can readily be applied to other cell lines, making it possible to address autophagy-related questions in different cellular systems without much effort. We tested the essential prerequisites for the suitability of Cyto-ID for cell sorting (Additional file 5: Fig. S5). Its temperature-dependent fluorescence intensity is of particular relevance: its robust stability at 4 °C for at least 90 min permits sorting (which may take an hour to complete), while its rapid decay at 37 °C allows Cyto-ID measurements at later timepoints without risk of interference from the Cyto-ID used for sorting.

The first noteworthy result obtained from cell sorting was the differential expression of *ULK1* in the fractions of MOLM-13 cells. Gump et al. concluded from their investigations that differences in gene expression were not responsible for differences in autophagy [70], whereas our observation suggests that fluctuations in autophagy can be related to fluctuations in gene expression, depending on the cell line investigated. *ULK1* expression level was even still significantly higher 24 h after sorting in Aut^HI^ relative to Aut^ME^ and Aut^LO^, thus matching still stronger autophagy in Aut^HI^ after this period. The homogeneous *ULK1* expression in sorted Jurkat cells, however, shows that *ULK1* expression heterogeneity is not a necessary precondition for cell-to-cell variations in autophagy. In any case, the Cyto-ID intensity-correlated abundance of *ULK1* mRNA in MOLM-13 cells in addition further supports the validity of cell sorting on the basis of Cyto-ID.

Our second remarkable finding was the clear association of autophagic and metabolic activity. Cancer cells often have elevated levels of constitutive autophagy thought to provide the metabolic building blocks, such as amino acids and lipids, required for proliferation [63–65]. We found lowest basal respiration and lowest ATP production as well as lowest baseline glycolysis in Aut^LO^ and, vice versa, highest bioenergetics parameters in Aut^HI^. These data are thus in keeping with the ability of autophagy to fuel the metabolism—both oxidative metabolism and aerobic glycolysis—of cancer cells. Yet they are also compatible with a reciprocal interrelation between autophagy and metabolism, with the latter governing the former [73] (which would imply that the cell-to-cell fluctuations in basal autophagy were a consequence of cell-to-cell fluctuations in metabolism). That said, a bidirectional relationship between autophagy and metabolism is conceivable, too. In any case, our results support the tightly coordinated action of autophagy and metabolism [63–65].

Finally, cell cycle analysis of Cyto-ID-sorted cells confirmed that the fraction with highest autophagy was predominantly present in the G2/M phase, a result that was further corroborated by gene and protein expression analyses of G2/M markers in sorted cells. This observation is in line with previous publications that demonstrated the relevance of autophagy in late stages of the cell cycle. Autophagy was found to promote the degradation of RHOA during cytokinesis, thereby maintaining genomic stability [74]. A study in budding yeast cells revealed a role of autophagy in the suppression of abnormal mitosis [75]. Another report showed that autophagy serves to degrade cyclin A2 during mitosis [76].

## Conclusions

To sum up, we have here provided clear evidence of high autophagy in G2/M-phase cells. We have accomplished that by establishing two new Cyto-ID-based methods, in particular Cyto-ID-based cell sorting. A promising next step in the understanding of the interaction of autophagy and cell cycle would be to explore what these results could mean for the therapeutic targeting of autophagy in cancer.

## Supplementary Information

**Additional file 1: Figure S1.** Autophagy inhibition affects etoposide-induced cell cycle arrest. Cells were exposed to etoposide with or without 3-MA for 24 h (Jurkat) or 48 h (MOLM-13). Cell cycle phases were determined by flow-cytometric analysis of DRAQ5-stained cells. Means ± SEM of each three separate measurements are shown.

**Additional file 2: Figure S2.** Chloroquine enhances etoposide-induced increase in Cyto-ID fluorescence intensity. After a 24-h treatment with etoposide, cells were exposed to 10 µM (Jurkat) or 25 µM (MOLM-13) chloroquine for the indicated times. Autophagy was determined by flow-cytometric analysis of Cyto-ID-stained cells. Cyto-ID fluorescence intensities were normalized to the mean Cyto-ID fluorescence intensities of untreated cells. Means ± SEM of each four separate measurements are shown (etoposide versus control: *P < 0.05; etoposide plus chloroquine versus etoposide without chloroquine: #P < 0.05).

**Additional file 3: Figure S3.** Cell cycle phase and autophagy are tightly associated. Autophagy and cell cycle phases were determined by flow-cytometric analysis of Cyto-ID + DRAQ5 double-stained cells. (A) Cell populations were grouped into quintiles based on their Cyto-ID fluorescence intensities. (B) Cell cycle histograms of each quintile of cells. Dot plots and histograms are representative of three independent measurements.

**Additional file 4: Figure S4.** Gating strategy for Cyto-ID-based cell sorting. Debris and aggregates were excluded from the sorting using a sequential gating strategy relying on FSC-A versus SSC-A followed by FSC-H versus FSC-W and SSC-H versus SSC-W. Dead cells were excluded by gating on Sytox Blue-negative cells. Cells were sorted into three subpopulations of approximately equal number based on their Cyto-ID fluorescence intensities, i.e., into populations with low, medium and high Cyto-ID fluorescence. The numbers within the plots indicate the percentages of the respective parent population

**Additional file 5: Figure S5.** Prerequisites for Cyto-ID-based cell sorting. (A) Toxicity of Cyto-ID. Cells were incubated with Cyto-ID at a dilution of 1:1000 for 4 h. Cell death was determined by flow-cytometric analysis of PI uptake. (B) Stability of Cyto-ID fluorescence. Cells were incubated with Cyto-ID at a dilution of 1:1000 at 4, 23 and 37 °C for the indicated times. Autophagy was determined by flow-cytometric analysis of Cyto-ID-stained cells. (C) Effect of sorting on Cyto-ID fluorescence. Autophagy of unsorted and Cyto-ID-sorted cells was determined by flow-cytometric analysis of Cyto-ID-stained cells approximately 1 h after sorting. Cyto-ID fluorescence intensities were normalized to the mean Cyto-ID fluorescence intensities of unsorted cells. (D) Relationship of cell size and Cyto-ID fluorescence intensity. Cell size is proportional to FSC, autophagy was determined by flow-cytometric analysis of Cyto-ID-stained cells. Dot plots are representative of three independent measurements. Cyto-ID fluorescence intensities and FSC values were normalized to the mean Cyto-ID fluorescence intensities and mean FSC values, respectively, of "low Cyto-ID" cells. Means ± SEM of each three or two (B, MOLM-13) separate measurements are shown.

**Additional file 6: Figure S6.** Metabolic phenotype of Cyto-ID-sorted cells. Cells were flow-cytometrically sorted on the basis of their Cyto-ID fluorescence intensity into subpopulations with low, medium and high Cyto-ID fluorescence (AutLO, AutME and AutHI, respectively). Oxygen consumption (OCR) and extracellular acidification rates (ECAR) were measured at basal conditions and after sequential injection of oligomycin (ATP synthase inhibitor), 2,4-dinitrophenol (DNP; oxidative phosphorylation uncoupler) and antimycin A (cytochrome c reductase inhibitor). (A) Higher basal mitochondrial respiration and ATP production in AutHI compared with AutLO and AutME. OCR as a proxy for oxidative metabolism was measured with the Seahorse Cell Mito Stress Test. (B) Higher glycolytic activity in AutHI compared with AutLO and AutME. ECAR was measured as a proxy for glycolytic activity. OCR and ECAR curves are representative of three independent measurements; each curve consisted of six readings.

**Additional file 7: Figure S7.** Autophagy in DRAQ5-sorted cells. Cells were flow-cytometrically sorted on the basis of their DRAQ5 fluorescence intensity into G1, S and G2/M phase cells. Autophagy was determined by flow-cytometric analysis of Cyto-ID-stained cells. Cyto-ID fluorescence intensities were normalized to the Cyto-ID fluorescence intensities of G1 phase cells. Means ± SEM of each three separate measurements are shown (*P < 0.05).

**Additional file 8: Figure S8.** Full immunoblot images. Black boxes indicate the cropped portion of each immunoblot shown in the corresponding main figures.

## Data Availability

All data generated or analyzed during this study are included in this article and its additional files.

## Abbreviations

3-MA: 3-Methyladenine
ATG: Autophagy-related
Aut^HI^: Cells with high autophagy
Aut^LO^: Cells with low autophagy
Aut^ME^: Cells with medium autophagy
CDK: Cyclin-dependent kinase
CQ: Chloroquine
ECAR: Extracellular acidification rate
FSC-A: Forward light scatter area
FSC-H: Forward light scatter height
FSC-W: Forward light scatter width
IFM: Indicator-free medium
OCR: Oxygen consumption rate
PI: Propidium iodide
SSC-A: Sideward light scatter area
SSC-H: Sideward light scatter height
SSC-W: Sideward light scatter width
