# Supplementary material for: Tight association of autophagy and cell cycle in leukemia cells
Source: Cell Mol Biol Lett. 2022 Apr 5;27:32. doi: 10.1186/s11658-022-00334-8 (PMC8981689; doi:10.1186/s11658-022-00334-8)
Supplement: Supplementary file 1 — Additional file 1: Figure S1. Autophagy inhibition affects etoposide-induced cell cycle arrest. Cells were exposed to etoposide with or without 3-MA for 24 h (Jurkat) or 48 h (MOLM-13). Cell cycle phases were determined by flow-cytometric analysis of DRAQ5-stained cells. Means ± SEM of each three separate measurements are shown. [file 11658_2022_334_MOESM1_ESM.pptx]

## Slide 1
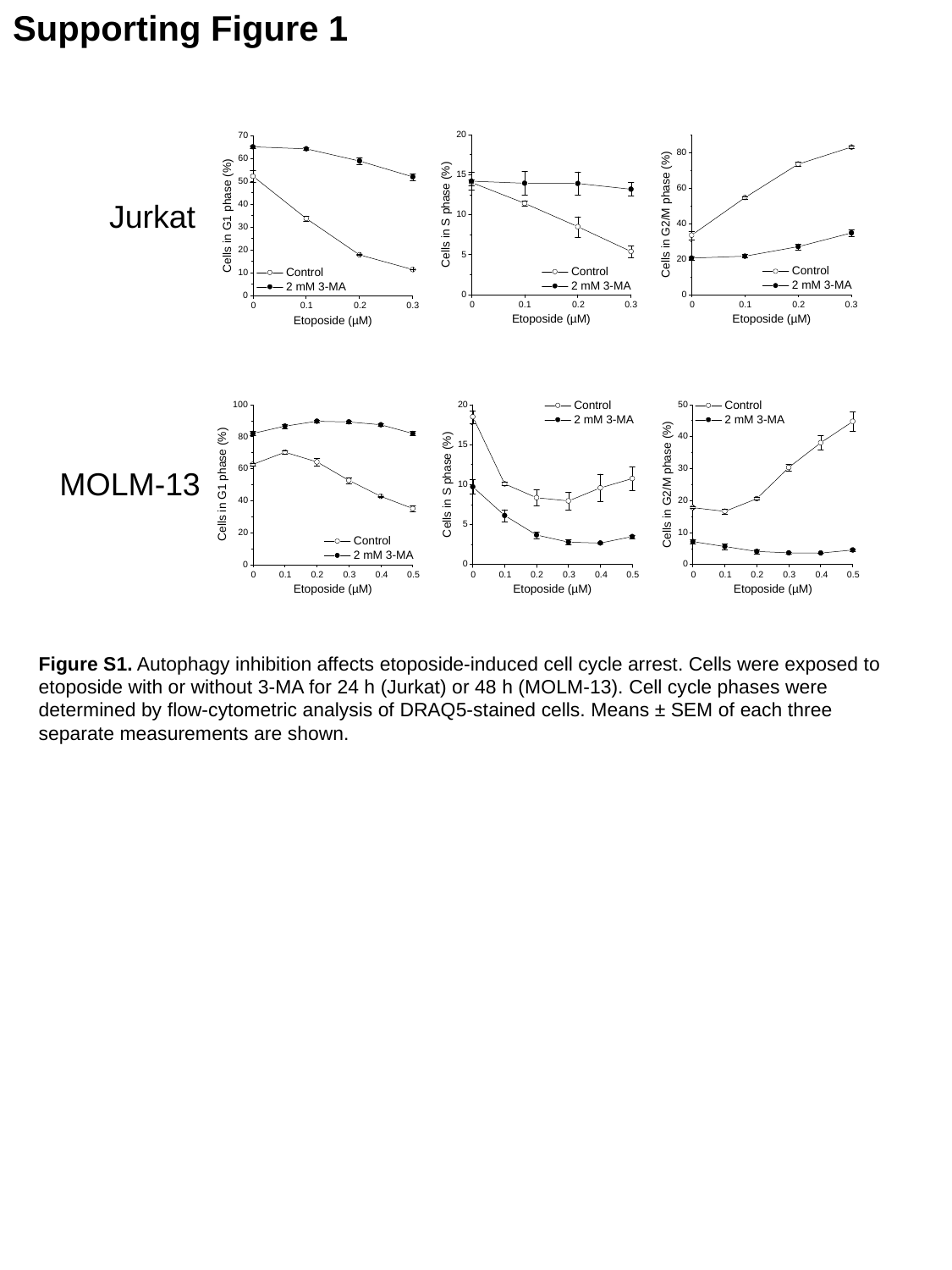

Supporting Figure 1
Jurkat
MOLM-13
Figure S1. Autophagy inhibition affects etoposide-induced cell cycle arrest. Cells were exposed to etoposide with or without 3-MA for 24 h (Jurkat) or 48 h (MOLM-13). Cell cycle phases were determined by flow-cytometric analysis of DRAQ5-stained cells. Means ± SEM of each three separate measurements are shown.
