# Supplementary material for: Tight association of autophagy and cell cycle in leukemia cells
Source: Cell Mol Biol Lett. 2022 Apr 5;27:32. doi: 10.1186/s11658-022-00334-8 (PMC8981689; doi:10.1186/s11658-022-00334-8)
Supplement: Supplementary file 2 — Additional file 2: Figure S2. Chloroquine enhances etoposide-induced increase in Cyto-ID fluorescence intensity. After a 24-h treatment with etoposide, cells were exposed to 10 µM (Jurkat) or 25 µM (MOLM-13) chloroquine for the indicated times. Autophagy was determined by flow-cytometric analysis of Cyto-ID-stained cells. Cyto-ID fluorescence intensities were normalized to the mean Cyto-ID fluorescence intensities of untreated cells. Means ± SEM of each four separate measurements are shown (etoposide versus control: *P < 0.05; etoposide plus chloroquine versus etoposide without chloroquine: #P < 0.05). [file 11658_2022_334_MOESM2_ESM.pptx]

## Slide 1
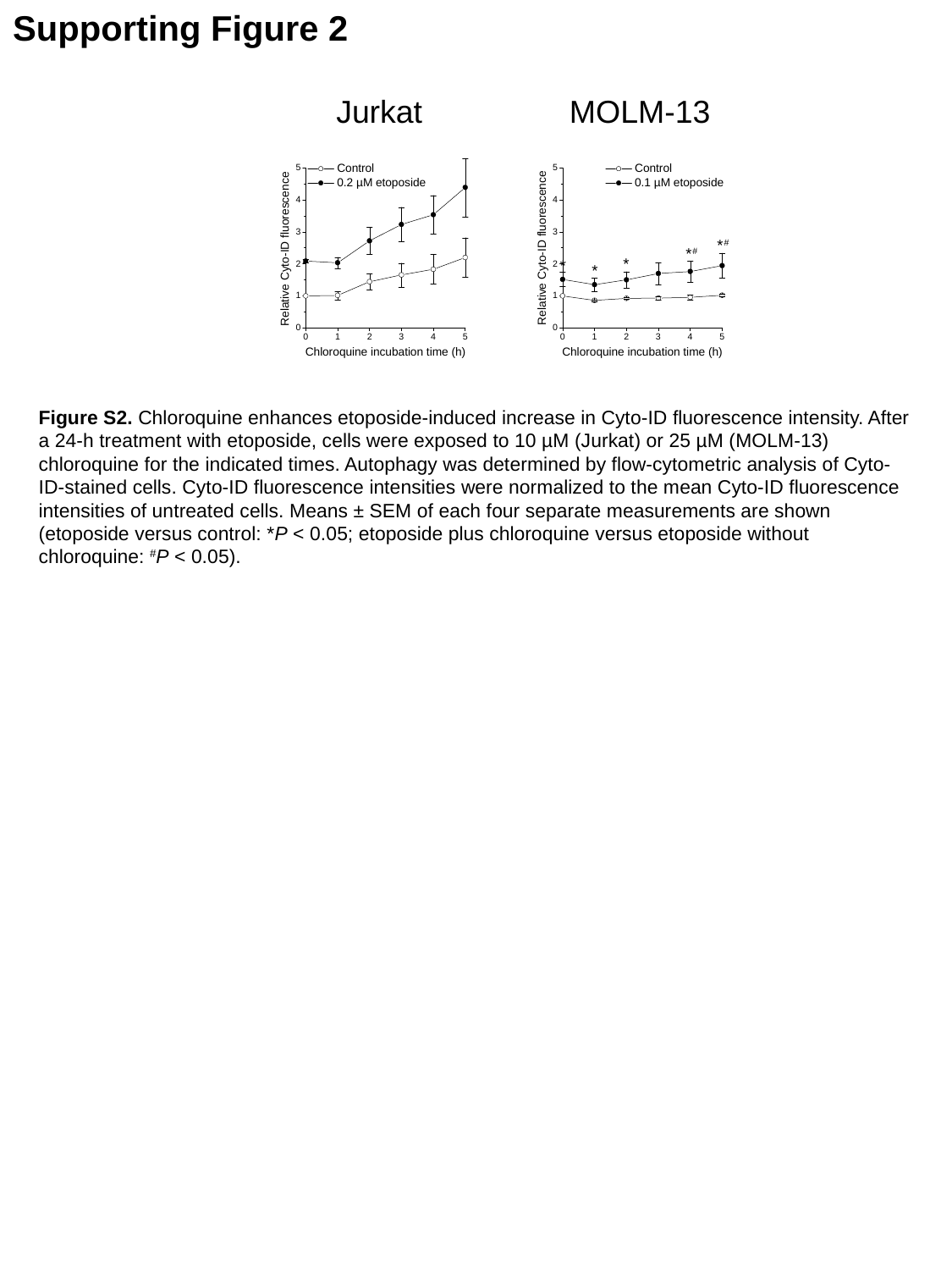

Supporting Figure 2
Jurkat
MOLM-13
Figure S2. Chloroquine enhances etoposide-induced increase in Cyto-ID fluorescence intensity. After a 24-h treatment with etoposide, cells were exposed to 10 µM (Jurkat) or 25 µM (MOLM-13) chloroquine for the indicated times. Autophagy was determined by flow-cytometric analysis of Cyto-ID-stained cells. Cyto-ID fluorescence intensities were normalized to the mean Cyto-ID fluorescence intensities of untreated cells. Means ± SEM of each four separate measurements are shown (etoposide versus control: *P < 0.05; etoposide plus chloroquine versus etoposide without chloroquine: #P < 0.05).
