# Supplementary material for: Tight association of autophagy and cell cycle in leukemia cells
Source: Cell Mol Biol Lett. 2022 Apr 5;27:32. doi: 10.1186/s11658-022-00334-8 (PMC8981689; doi:10.1186/s11658-022-00334-8)
Supplement: Supplementary file 3 — Additional file 3: Figure S3. Cell cycle phase and autophagy are tightly associated. Autophagy and cell cycle phases were determined by flow-cytometric analysis of Cyto-ID + DRAQ5 double-stained cells. (A) Cell populations were grouped into quintiles based on their Cyto-ID fluorescence intensities. (B) Cell cycle histograms of each quintile of cells. Dot plots and histograms are representative of three independent measurements. [file 11658_2022_334_MOESM3_ESM.pptx]

## Slide 1
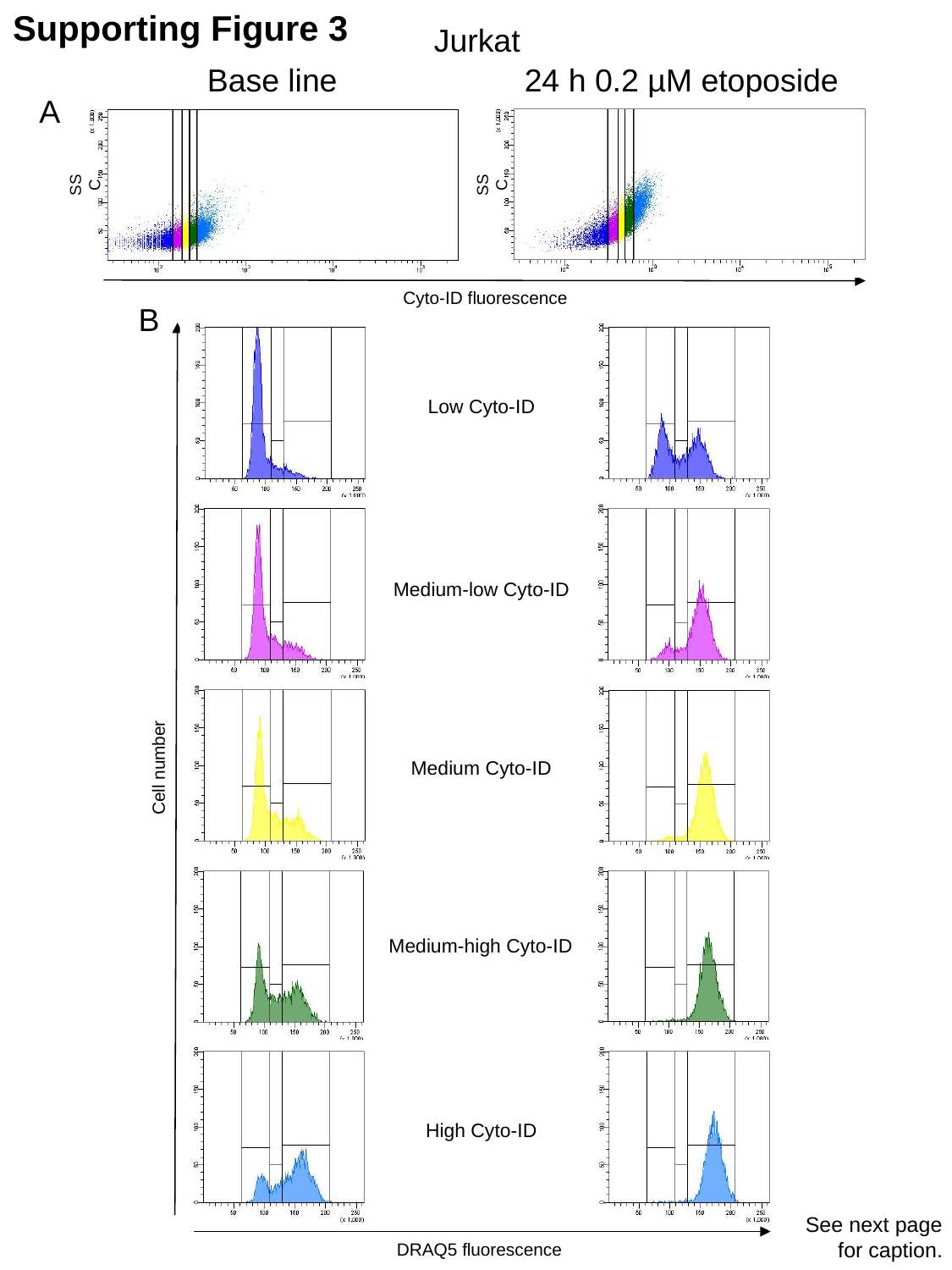

Supporting Figure 3
Jurkat
Base line
24 h 0.2 µM etoposide
A
SSC
SSC
Cyto-ID fluorescence
B
Low Cyto-ID
Medium-low Cyto-ID
Cell number
Medium Cyto-ID
Medium-high Cyto-ID
High Cyto-ID
See next page
for caption.
DRAQ5 fluorescence

## Slide 2
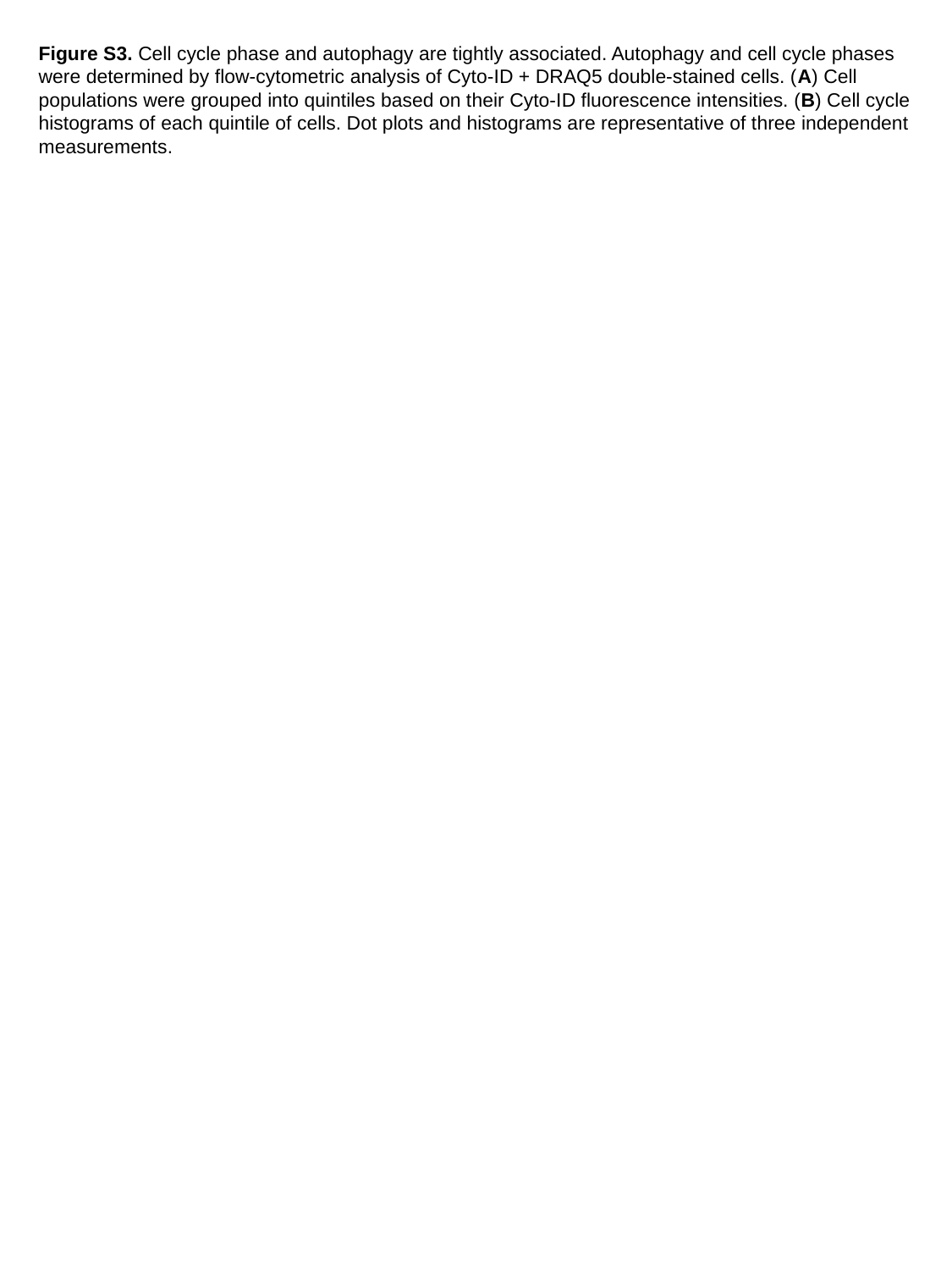

Figure S3. Cell cycle phase and autophagy are tightly associated. Autophagy and cell cycle phases were determined by flow-cytometric analysis of Cyto-ID + DRAQ5 double-stained cells. (A) Cell populations were grouped into quintiles based on their Cyto-ID fluorescence intensities. (B) Cell cycle histograms of each quintile of cells. Dot plots and histograms are representative of three independent measurements.
