# Supplementary material for: Tight association of autophagy and cell cycle in leukemia cells
Source: Cell Mol Biol Lett. 2022 Apr 5;27:32. doi: 10.1186/s11658-022-00334-8 (PMC8981689; doi:10.1186/s11658-022-00334-8)
Supplement: Supplementary file 4 — Additional file 4: Figure S4. Gating strategy for Cyto-ID-based cell sorting. Debris and aggregates were excluded from the sorting using a sequential gating strategy relying on FSC-A versus SSC-A followed by FSC-H versus FSC-W and SSC-H versus SSC-W. Dead cells were excluded by gating on Sytox Blue-negative cells. Cells were sorted into three subpopulations of approximately equal number based on their Cyto-ID fluorescence intensities, i.e., into populations with low, medium and high Cyto-ID fluorescence. The numbers within the plots indicate the percentages of the respective parent population [file 11658_2022_334_MOESM4_ESM.pptx]

## Slide 1
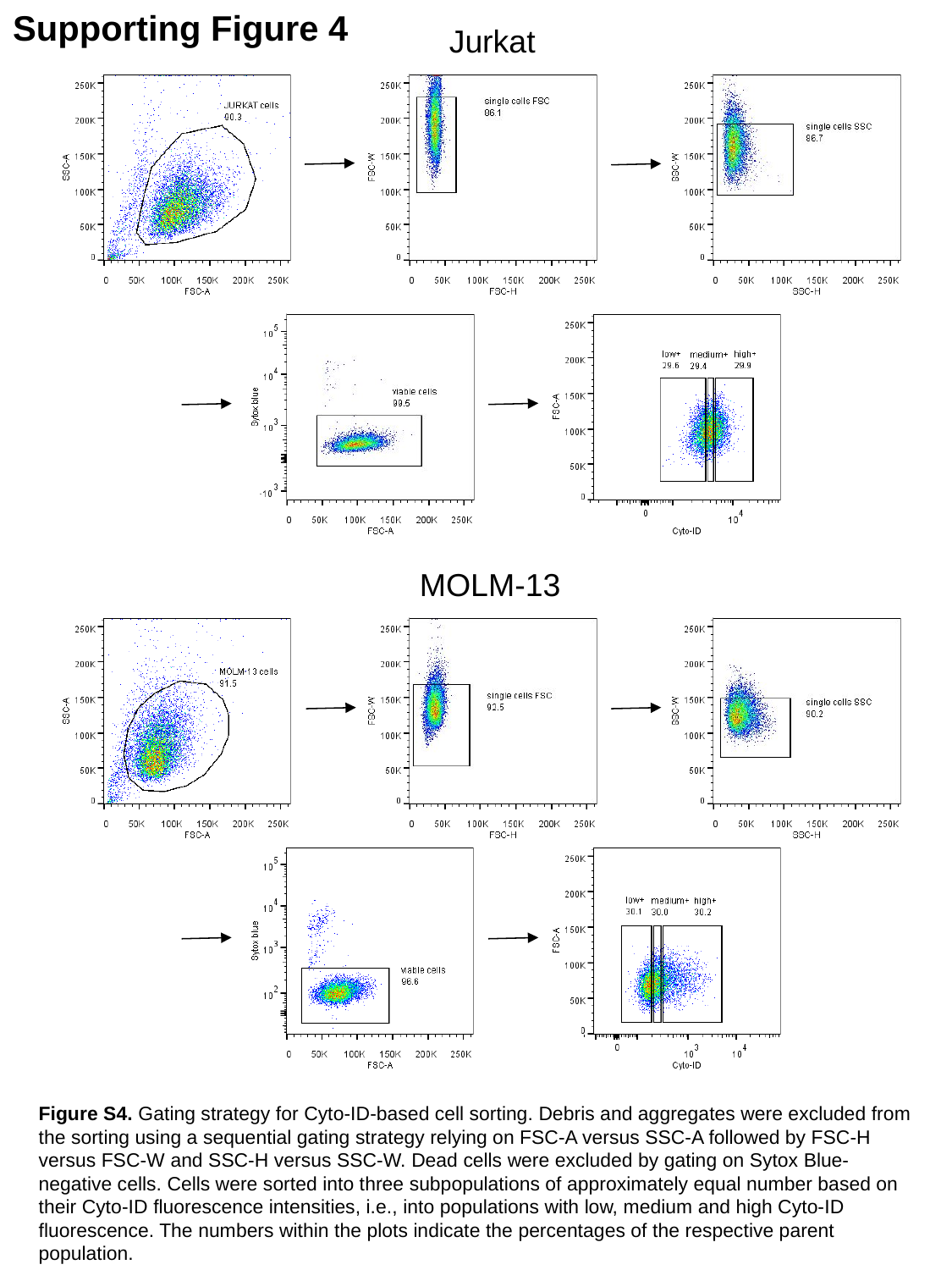

Supporting Figure 4
Jurkat
MOLM-13
Figure S4. Gating strategy for Cyto-ID-based cell sorting. Debris and aggregates were excluded from the sorting using a sequential gating strategy relying on FSC-A versus SSC-A followed by FSC-H versus FSC-W and SSC-H versus SSC-W. Dead cells were excluded by gating on Sytox Blue-negative cells. Cells were sorted into three subpopulations of approximately equal number based on their Cyto-ID fluorescence intensities, i.e., into populations with low, medium and high Cyto-ID fluorescence. The numbers within the plots indicate the percentages of the respective parent population.
