# Supplementary material for: Tight association of autophagy and cell cycle in leukemia cells
Source: Cell Mol Biol Lett. 2022 Apr 5;27:32. doi: 10.1186/s11658-022-00334-8 (PMC8981689; doi:10.1186/s11658-022-00334-8)
Supplement: Supplementary file 5 — Additional file 5: Figure S5. Prerequisites for Cyto-ID-based cell sorting. (A) Toxicity of Cyto-ID. Cells were incubated with Cyto-ID at a dilution of 1:1000 for 4 h. Cell death was determined by flow-cytometric analysis of PI uptake. (B) Stability of Cyto-ID fluorescence. Cells were incubated with Cyto-ID at a dilution of 1:1000 at 4, 23 and 37 °C for the indicated times. Autophagy was determined by flow-cytometric analysis of Cyto-ID-stained cells. (C) Effect of sorting on Cyto-ID fluorescence. Autophagy of unsorted and Cyto-ID-sorted cells was determined by flow-cytometric analysis of Cyto-ID-stained cells approximately 1 h after sorting. Cyto-ID fluorescence intensities were normalized to the mean Cyto-ID fluorescence intensities of unsorted cells. (D) Relationship of cell size and Cyto-ID fluorescence intensity. Cell size is proportional to FSC, autophagy was determined by flow-cytometric analysis of Cyto-ID-stained cells. Dot plots are representative of three independent measurements. Cyto-ID fluorescence intensities and FSC values were normalized to the mean Cyto-ID fluorescence intensities and mean FSC values, respectively, of "low Cyto-ID" cells. Means ± SEM of each three or two (B, MOLM-13) separate measurements are shown. [file 11658_2022_334_MOESM5_ESM.pptx]

## Slide 1
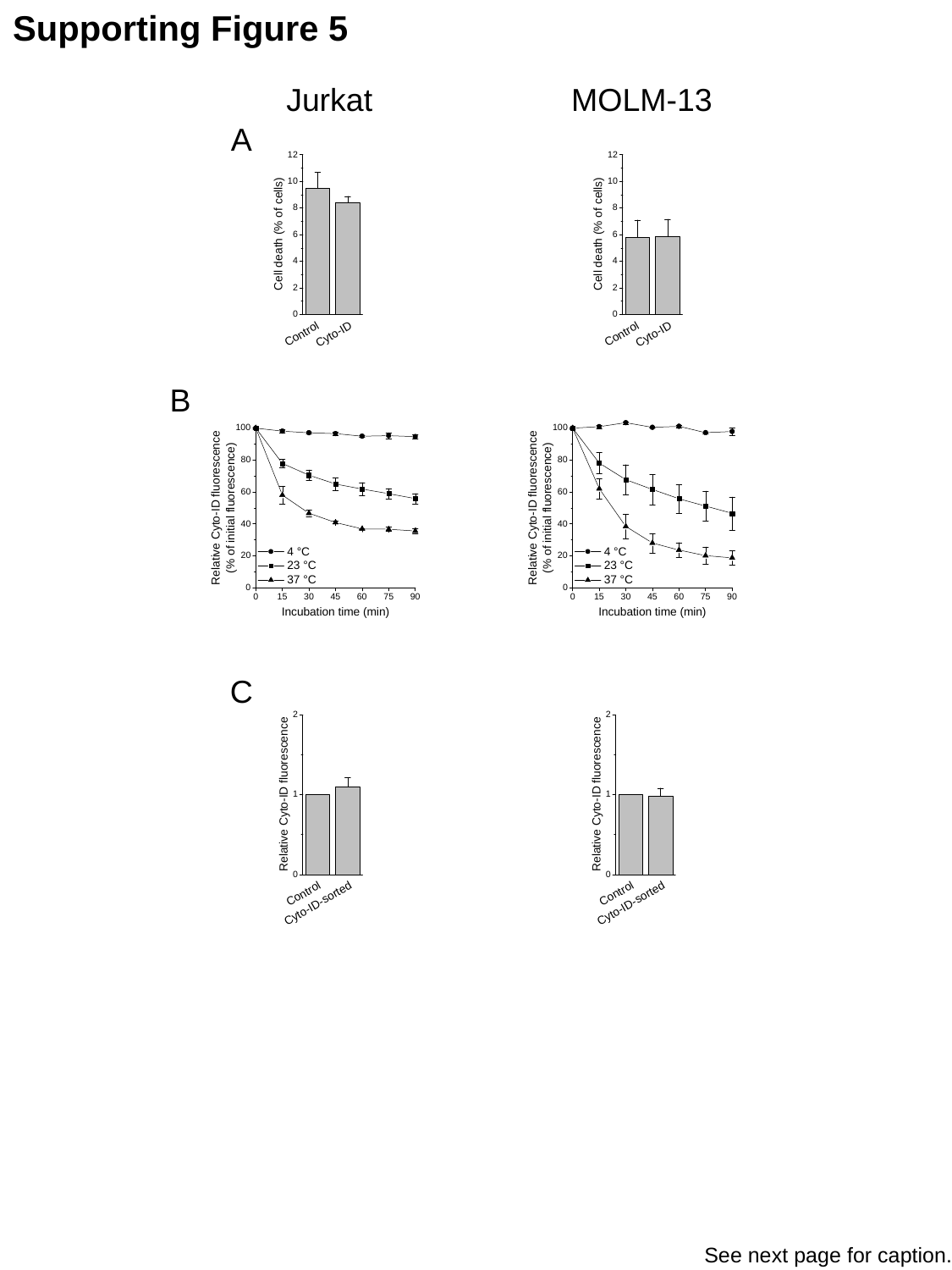

Supporting Figure 5
Jurkat
MOLM-13
A
B
C
See next page for caption.

## Slide 2
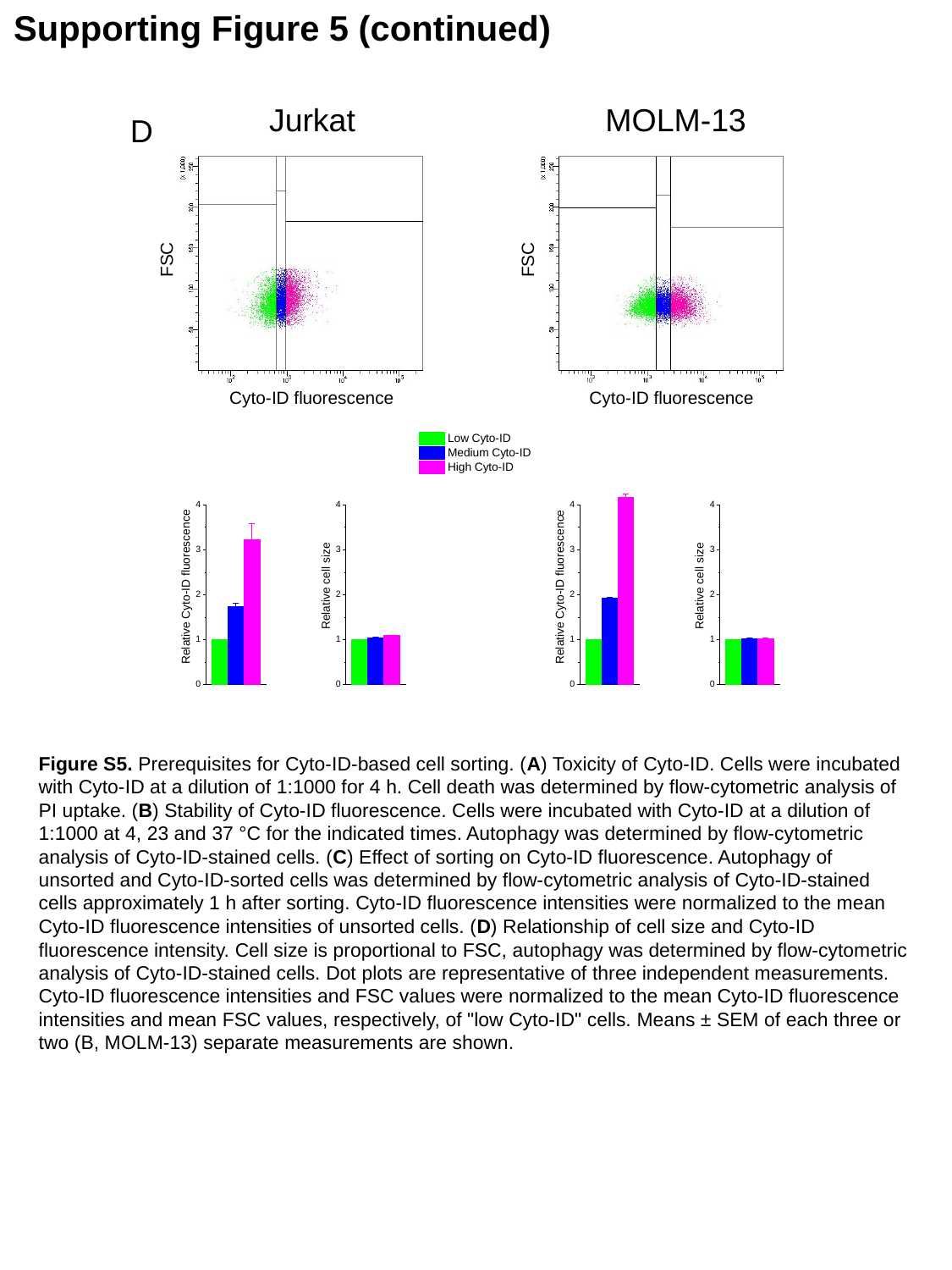

Supporting Figure 5 (continued)
Jurkat
MOLM-13
D
FSC
FSC
Cyto-ID fluorescence
Cyto-ID fluorescence
Figure S5. Prerequisites for Cyto-ID-based cell sorting. (A) Toxicity of Cyto-ID. Cells were incubated with Cyto-ID at a dilution of 1:1000 for 4 h. Cell death was determined by flow-cytometric analysis of PI uptake. (B) Stability of Cyto-ID fluorescence. Cells were incubated with Cyto-ID at a dilution of 1:1000 at 4, 23 and 37 °C for the indicated times. Autophagy was determined by flow-cytometric analysis of Cyto-ID-stained cells. (C) Effect of sorting on Cyto-ID fluorescence. Autophagy of unsorted and Cyto-ID-sorted cells was determined by flow-cytometric analysis of Cyto-ID-stained cells approximately 1 h after sorting. Cyto-ID fluorescence intensities were normalized to the mean Cyto-ID fluorescence intensities of unsorted cells. (D) Relationship of cell size and Cyto-ID fluorescence intensity. Cell size is proportional to FSC, autophagy was determined by flow-cytometric analysis of Cyto-ID-stained cells. Dot plots are representative of three independent measurements. Cyto-ID fluorescence intensities and FSC values were normalized to the mean Cyto-ID fluorescence intensities and mean FSC values, respectively, of "low Cyto-ID" cells. Means ± SEM of each three or two (B, MOLM-13) separate measurements are shown.
