# Supplementary material for: Tight association of autophagy and cell cycle in leukemia cells
Source: Cell Mol Biol Lett. 2022 Apr 5;27:32. doi: 10.1186/s11658-022-00334-8 (PMC8981689; doi:10.1186/s11658-022-00334-8)
Supplement: Supplementary file 6 — Additional file 6: Figure S6. Metabolic phenotype of Cyto-ID-sorted cells. Cells were flow-cytometrically sorted on the basis of their Cyto-ID fluorescence intensity into subpopulations with low, medium and high Cyto-ID fluorescence (AutLO, AutME and AutHI, respectively). Oxygen consumption (OCR) and extracellular acidification rates (ECAR) were measured at basal conditions and after sequential injection of oligomycin (ATP synthase inhibitor), 2,4-dinitrophenol (DNP; oxidative phosphorylation uncoupler) and antimycin A (cytochrome c reductase inhibitor). (A) Higher basal mitochondrial respiration and ATP production in AutHI compared with AutLO and AutME. OCR as a proxy for oxidative metabolism was measured with the Seahorse Cell Mito Stress Test. (B) Higher glycolytic activity in AutHI compared with AutLO and AutME. ECAR was measured as a proxy for glycolytic activity. OCR and ECAR curves are representative of three independent measurements; each curve consisted of six readings. [file 11658_2022_334_MOESM6_ESM.pptx]

## Slide 1
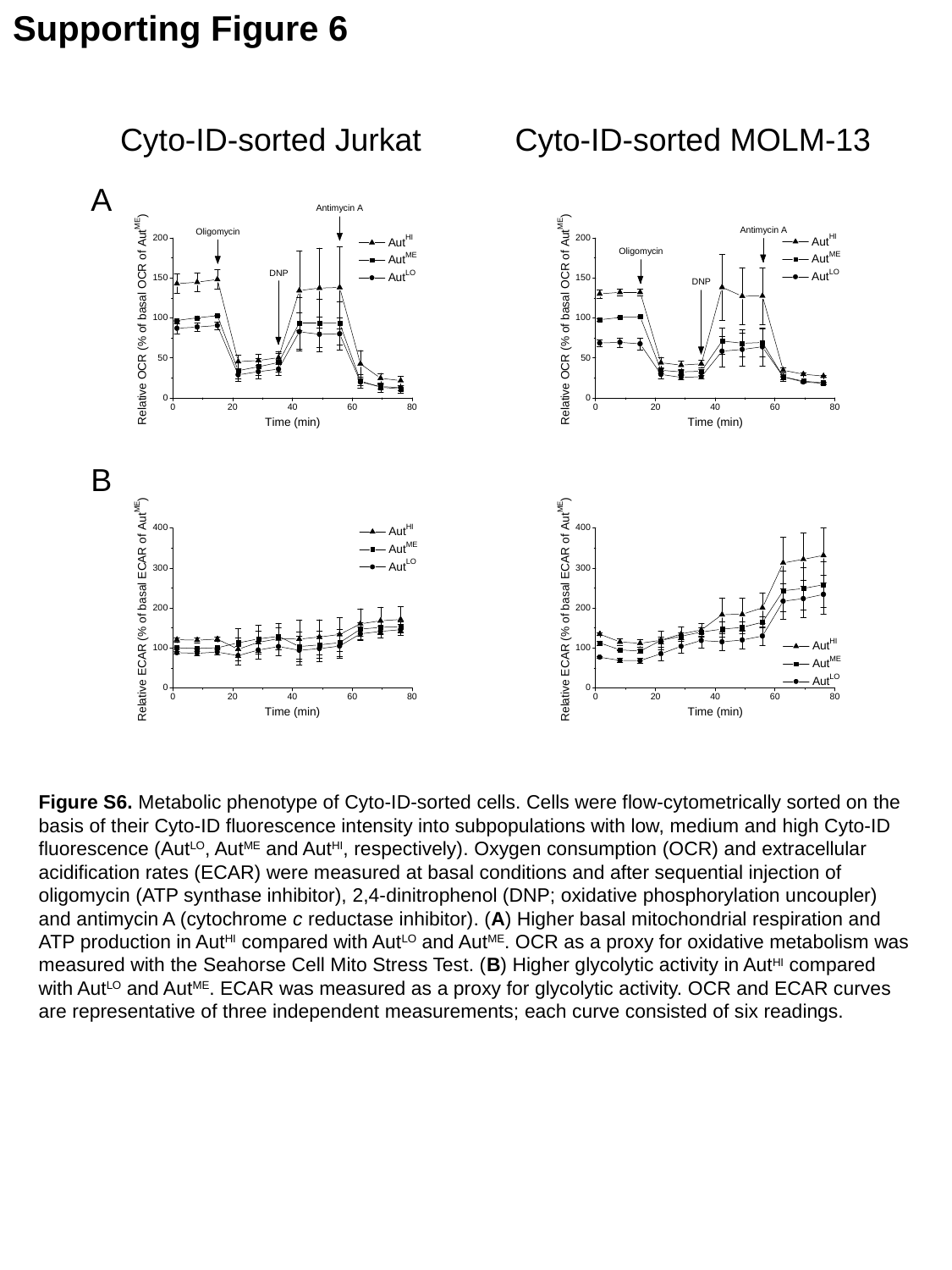

Supporting Figure 6
Cyto-ID-sorted Jurkat
Cyto-ID-sorted MOLM-13
A
B
Figure S6. Metabolic phenotype of Cyto-ID-sorted cells. Cells were flow-cytometrically sorted on the basis of their Cyto-ID fluorescence intensity into subpopulations with low, medium and high Cyto-ID fluorescence (AutLO, AutME and AutHI, respectively). Oxygen consumption (OCR) and extracellular acidification rates (ECAR) were measured at basal conditions and after sequential injection of oligomycin (ATP synthase inhibitor), 2,4-dinitrophenol (DNP; oxidative phosphorylation uncoupler) and antimycin A (cytochrome c reductase inhibitor). (A) Higher basal mitochondrial respiration and ATP production in AutHI compared with AutLO and AutME. OCR as a proxy for oxidative metabolism was measured with the Seahorse Cell Mito Stress Test. (B) Higher glycolytic activity in AutHI compared with AutLO and AutME. ECAR was measured as a proxy for glycolytic activity. OCR and ECAR curves are representative of three independent measurements; each curve consisted of six readings.
