# Supplementary material for: Tight association of autophagy and cell cycle in leukemia cells
Source: Cell Mol Biol Lett. 2022 Apr 5;27:32. doi: 10.1186/s11658-022-00334-8 (PMC8981689; doi:10.1186/s11658-022-00334-8)
Supplement: Supplementary file 7 — Additional file 7: Figure S7. Autophagy in DRAQ5-sorted cells. Cells were flow-cytometrically sorted on the basis of their DRAQ5 fluorescence intensity into G1, S and G2/M phase cells. Autophagy was determined by flow-cytometric analysis of Cyto-ID-stained cells. Cyto-ID fluorescence intensities were normalized to the Cyto-ID fluorescence intensities of G1 phase cells. Means ± SEM of each three separate measurements are shown (*P < 0.05). [file 11658_2022_334_MOESM7_ESM.pptx]

## Slide 1
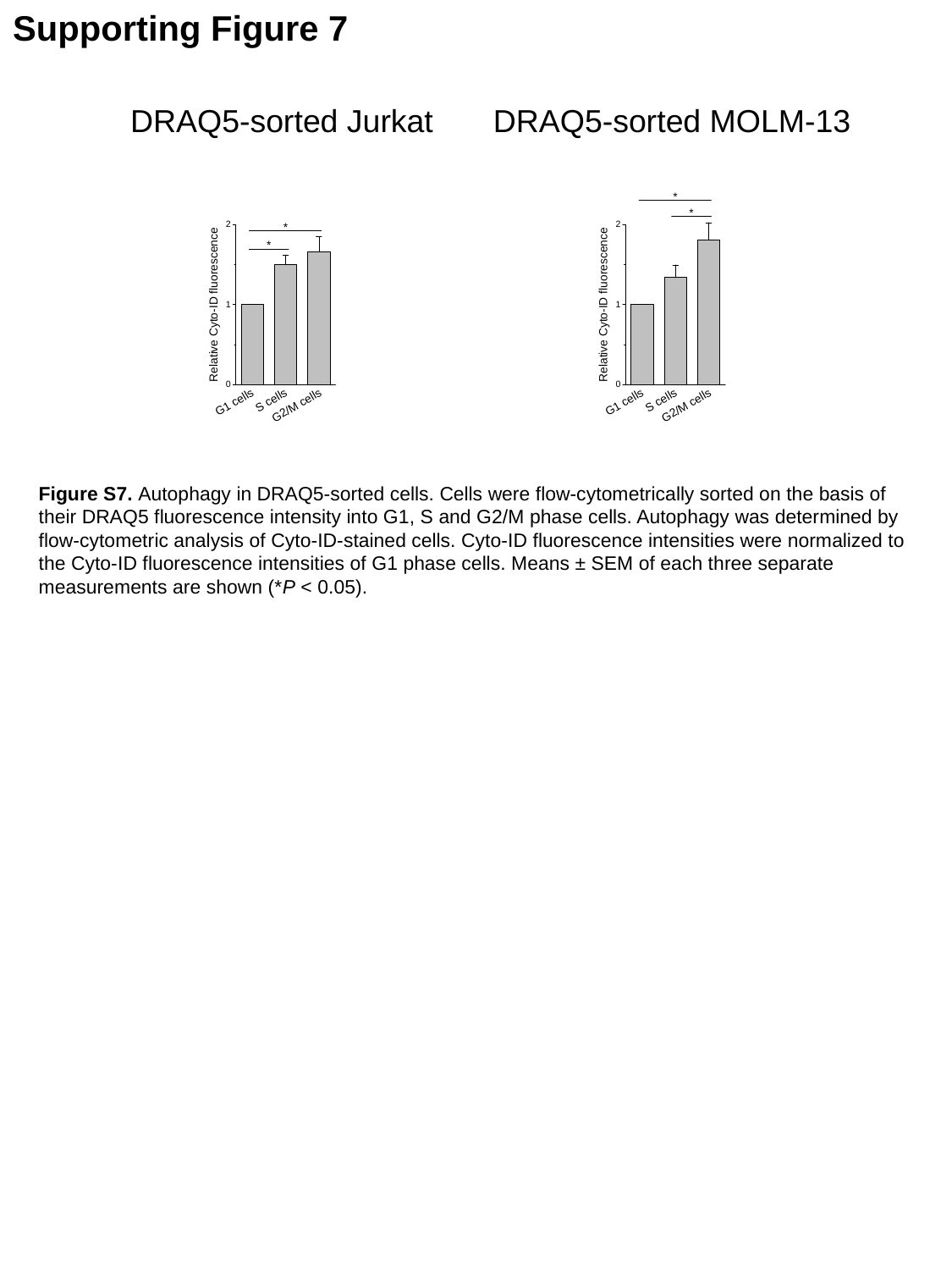

Supporting Figure 7
DRAQ5-sorted Jurkat
DRAQ5-sorted MOLM-13
Figure S7. Autophagy in DRAQ5-sorted cells. Cells were flow-cytometrically sorted on the basis of their DRAQ5 fluorescence intensity into G1, S and G2/M phase cells. Autophagy was determined by flow-cytometric analysis of Cyto-ID-stained cells. Cyto-ID fluorescence intensities were normalized to the Cyto-ID fluorescence intensities of G1 phase cells. Means ± SEM of each three separate measurements are shown (*P < 0.05).
