# Supplementary material for: Tight association of autophagy and cell cycle in leukemia cells
Source: Cell Mol Biol Lett. 2022 Apr 5;27:32. doi: 10.1186/s11658-022-00334-8 (PMC8981689; doi:10.1186/s11658-022-00334-8)
Supplement: Supplementary file 8 — Additional file 8: Figure S8. Full immunoblot images. Black boxes indicate the cropped portion of each immunoblot shown in the corresponding main figures. [file 11658_2022_334_MOESM8_ESM.pptx]

## Slide 1
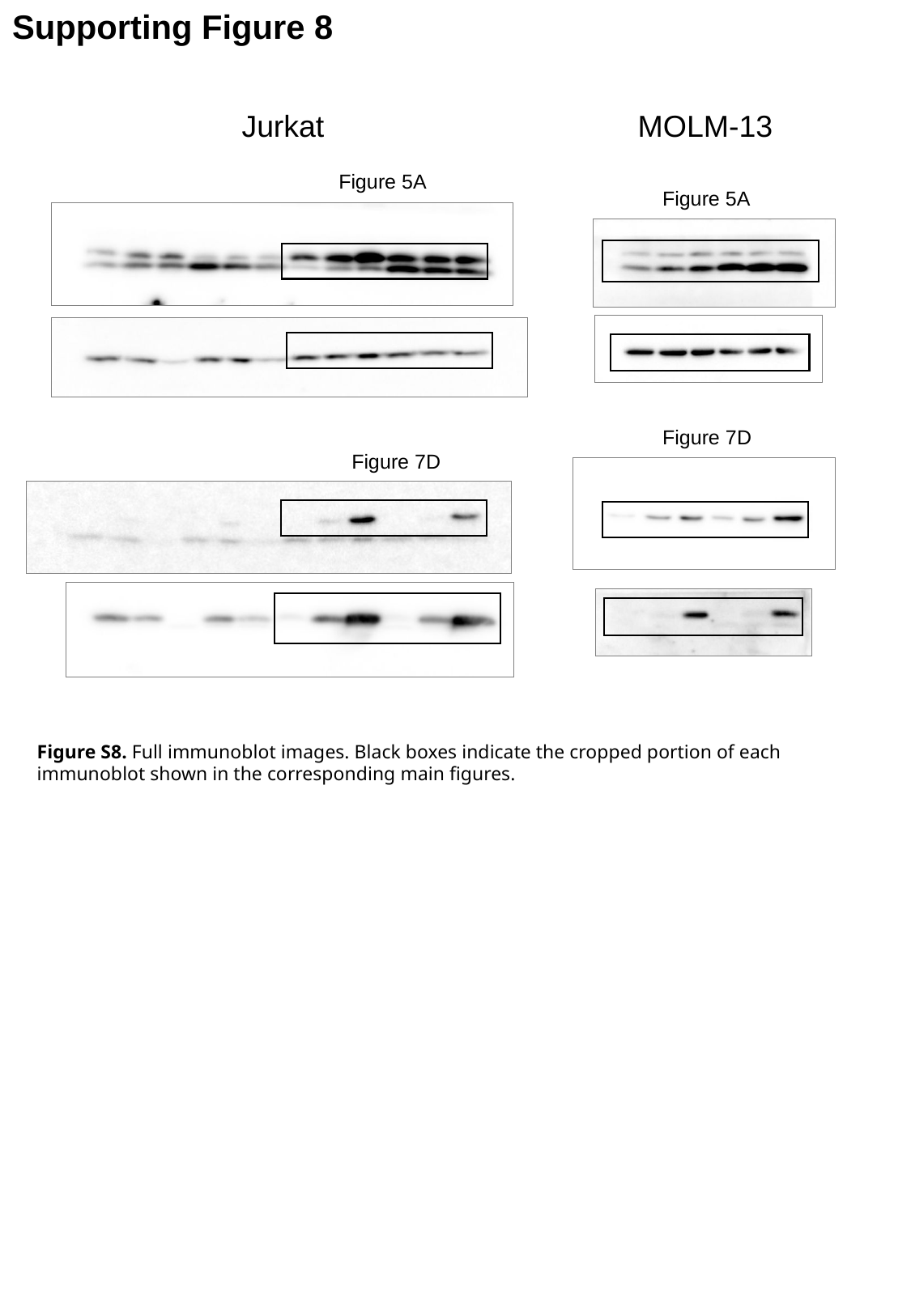

Supporting Figure 8
MOLM-13
Jurkat
Figure 5A
Figure 5A
Figure 7D
Figure 7D
Figure S8. Full immunoblot images. Black boxes indicate the cropped portion of each immunoblot shown in the corresponding main figures.
